# Machine learning for population-level risk prediction of future cholangiocarcinoma

**DOI:** 10.1016/j.ebiom.2026.106433

**Published:** 2026-08-12

**Authors:** Felix van Haag, Jan Clusmann, Paul-Henry Koop, Ryan Goodson, Ilya Tryakin, Shun-Ichi Wakabayashi, Tobias Seibel, Niharika Jakhar, Helen Ye Rim Huang, David Y. Zhang, Inuk Zandvakili, Takefumi Kimura, Nobuharu Tamaki, Arunkumar Krishnan, Pedro Miguel Rodrigues, Jesus Maria Banales, Alessio Gerussi, Anna Saborowski, Daniel Rader, Jakob Nikolas Kather, Kai Markus Schneider, Carolin Victoria Schneider

**Affiliations:** a Department of Internal Medicine III, Gastroenterology, Metabolic Diseases and Intensive Care, University Hospital RWTH Aachen, Aachen, Germany; b Department of Medicine I, Faculty of Medicine and University Hospital Carl Gustav Carus, TUD Dresden University of Technology, Dresden, Germany; c Else Kroener Fresenius Centre for Digital Health, Faculty of Medicine and University Hospital Carl Gustav Carus, TUD Dresden University of Technology, Dresden, Germany; d German Cancer Consortium (DKTK) partner site Dresden and German Cancer Research Centre (DKFZ), Heidelberg, Germany; e College of Medicine, University of Cincinnati, Cincinnati, OH, 45267, USA; f Division of Gastroenterology, Department of Medicine, Shinshu University School of Medicine, Nagano, Japan; g The Perelman School of Medicine, The Institute for Translational Medicine and Therapeutics, University of Pennsylvania, Philadelphia, PA, USA; h Department of Medicine, University of Pennsylvania Perelman School of Medicine, Philadelphia, PA, 19104, USA; i Department of Genetics, University of Pennsylvania Perelman School of Medicine, Philadelphia, PA, 19104, USA; j Division of Gastroenterology and Hepatology, Department of Internal Medicine, College of Medicine, University of Cincinnati, Cincinnati, OH, USA; k Department of Gastroenterology and Hepatology, Musashino Red Cross Hospital, Tokyo, Japan; l Department of Supportive Oncology, Atrium Health Levine Cancer, Charlotte, NC, 28204, USA; m Department of Liver and Gastrointestinal Diseases, Biogipuzkoa Health Research Institute, Donostia University Hospital, University of the Basque Country (UPV/EHU), Ikerbasque, CIBERehd, San Sebastian, Spain; n Department of Biochemistry and Genetics, School of Sciences, University of Navarra, Pamplona, Spain; o Department of Medicine and Surgery, University of Milano-Bicocca, Monza, Italy; p Centre for Autoimmune Liver Diseases & Division of Gastroenterology, Fondazione IRCCS San Gerardo dei Tintori, ERN RARE LIVER, Monza, Italy; q Department of Gastroenterology, Hepatology, Infectious Diseases and Endocrinology, Hannover Medical School, Hannover, Germany; r Medical Oncology, National Centre for Tumor Diseases (NCT), University Hospital Heidelberg, Heidelberg, Germany; s Pathology & Data Analytics, Leeds Institute of Medical Research at St James’s, University of Leeds, Leeds, United Kingdom; t Centre for Regenerative Therapies Dresden (CRTD), Technische Universität (TU), Dresden, Germany; u Division of Gastroenterology and Hepatology, Department of Medicine, University of Pennsylvania, Philadelphia, PA, USA

**Keywords:** Predictive modelling, Machine learning, Big data, Cholangiocarcinoma, Risk stratification

## Abstract

**Background:**

The poor prognosis of cholangiocarcinoma (CCA) is largely driven by rapid, asymptomatic disease progression, which usually results in a late diagnosis in the absence of established screening strategies. An early, cost-effective, and universally applicable risk assessment strategy would therefore be valuable.

**Methods:**

We developed machine learning (ML) models on prospective, multimodal data from 487,495 UK Biobank (UKB) participants, of whom 649 developed CCA during follow-up. Data from England (80%) were utilised for ML development via five-fold cross-validation, and then all models were tested on withheld data from Scotland, Wales, and Newcastle (20%). Iterative ablation studies reduced inputs from >150 features across demographic data, lifestyle, health records, blood parameters, genomics, and metabolomics to models built on five and ten routinely available clinical parameters. These were externally validated in the Penn Medicine Biobank (PMBB; n = 2638; 28 CCA), All of Us Research Program (AOU; n = 330,433; 362 CCA), Japan Medical Data Centre Claims Database (JMDC; n = 8,425,522; 723 CCA) and TriNetX (n = 728,886; 1592 CCA).

**Findings:**

We show that ML models integrating biliary-disease associated health records and Gamma glutamyltransferase can stratify risk of future CCA. Evaluation on the UKB test set as well as three independent cohorts revealed robust performance and generalisability across ethnicities. We achieved AUROCs of 0.71 [95% CI: 0.703–0.711], 0.77 [95% CI: 0.764–0.778 ], 0.796 [95% CI: 0.795–0.798] and 0.8 [95% CI: 0.794–0.805] for UKB, PMBB, AOU, and JMDC respectively, with respective AUPRCs of 0.014 [95% CI: 0.009–0.018], 0.042 [95% CI: 0.037–0.048], 0.038 [95% CI: 0.033–0.042] and 0.001 [95% CI: 0.001–0.001]. In AOU, application of the Youden J-optimised threshold yielded a number needed to screen of 79. Separate models for intra- and extrahepatic CCA did not improve performance. In line with the pathophysiology, performance declined for longer intervals between assessment and event. A group-level analysis in the TriNetX cohort revealed hazard ratios of up to 82.5 [95% CI: 26.4–257.96]. We provide extensive interpretability results and release all source codes used to develop the presented models.

**Interpretation:**

We provide a comprehensive framework for early CCA risk stratification in the general population, identifying key predictors, and demonstrating the potential of data-driven models in personalised screening for hepatobiliary cancer.

**Funding:**

German Cancer Aid (grant #70115730), Junior Principal Investigator Fellowship programme of RWTH Aachen Excellence strategy.

**Research in context**

**Evidence before this study**

Cholangiocarcinoma (CCA) remains a rare and highly lethal malignancy, often diagnosed at advanced stages due to its asymptomatic progression, rendering curative surgical resection less feasible. The literature consistently identifies older age, parasitic infections (notably in endemic regions), and primary sclerosing cholangitis (PSC) as major risk factors, though most cases arise sporadically without identifiable predisposing conditions. Current clinical guidelines recommend routine surveillance only for individuals with PSC, leaving the majority of patients without access to structured early detection strategies.

**Added value of this study**

We provide a population-level risk score for cholangiocarcinoma (CCA) that is directly applicable in routine clinical settings. Our model uses widely available electronic health records and a single routinely measured laboratory parameter, enabling scalable risk stratification without relying on costly or invasive diagnostics. Through validation across three large, independent, and demographically distinct cohorts, we demonstrate consistent predictive performance and broad generalisability, supporting the potential for integration into population-level screening frameworks.

**Implications of all the available evidence**

Our ML-based risk score combines a single routinely measured blood biomarker with EHR and patient-reported data to enable low-cost, scalable risk stratification for CCA in the general population. This approach could serve as a triage step to identify individuals for further diagnostic evaluation with ultrasound or MR imaging, and could support dynamic reassessment over time. As a broad CCA prescreening tool, it can serve as a benchmark and provides a readily deployable framework that can be improved by emerging biomarkers or multimodal data in future studies. By addressing the current lack of early detection, it lays the groundwork for broader implementation in primary care and risk-adapted surveillance strategies.

## Introduction

Cholangiocarcinoma (CCA) is a highly aggressive bile duct malignancy, accounting for 3% of gastrointestinal cancers and ranking as the second most common malignant primary liver cancer after hepatocellular carcinoma (HCC).^1^ Current classifications distinguish intrahepatic (iCCA), perihilar (pCCA) and distal (dCCA) subtypes, whereas former ICD10 classifications separated only intrahepatic and extrahepatic subtypes.^2^, ^3^, ^4^, ^5^, ^6^ Prognosis remains poor across all subtypes,^7^, ^8^, ^9^ with median survival below 13 months in locally advanced or metastatic disease stages. However, outcomes vary depending on cancer subtype, surgical resectability, and, crucially, stage of cancer at diagnosis.^10^, ^11^, ^12^, ^13^, ^14^, ^15^

Most CCAs arise sporadically without identifiable predisposing factors.^16^ Established risks include older age, chronic biliary inflammation, including primary sclerosing cholangitis (PSC) and cholelithiasis, and endemic liver fluke infections.^1^^,^^8^^,^^16^, ^17^, ^18^, ^19^, ^20^ Alcohol consumption, smoking, obesity, and diabetes have been discussed as risk factors, albeit primarily for iCCA.^17^^,^^18^^,^^20^, ^21^, ^22^

Early-stage CCA is often asymptomatic, with 40% of patients with iCCA detected incidentally, and symptoms of CCA indicating advanced disease stages.^23^ In contrast, eCCA more commonly causes cholestatic symptoms (jaundice, pruritus, pale stools, dark urine), also detectable in earlier stages. Diagnosis relies on contrast-enhanced magnetic resonance imaging (MRI) or magnetic resonance cholangiopancreatography (MRCP), with confirmation via endoscopic retrograde cholangiopancreatography (ERCP) bile brush cytology or biopsy.^20^^,^^21^ Curative surgical treatment is feasible in only ∼35% of patients with CCA.^14^^,^^24^^,^^25^ Despite new immunotherapeutic treatment strategies for advanced stages,^26^ survival remains poor, highlighting the critical need for early detection strategies.^14^^,^^26^^,^^27^

Currently, population-wide screening is not feasible, given the low prevalence of CCA. Guidelines recommend targeted screening only for individuals with PSC, prior choledochal cyst resection, or cirrhosis.^2^^,^^16^^,^^20^^,^^21^ Affordable preselection tools or scores that quantify the individual risk and allow image-based screening for high-risk individuals are a promising, yet underexplored strategy for CCA screening.^20^^,^^21^^,^^28^^,^^29^ This emphasises the need for cost-efficient, broadly applicable risk stratification tools to prescreen patients and guide further diagnostic evaluation. Developing an affordable and practical risk assessment tool for use in daily clinical practice could improve early detection and ultimately patient prognosis.^30^

**Fig. 1:**
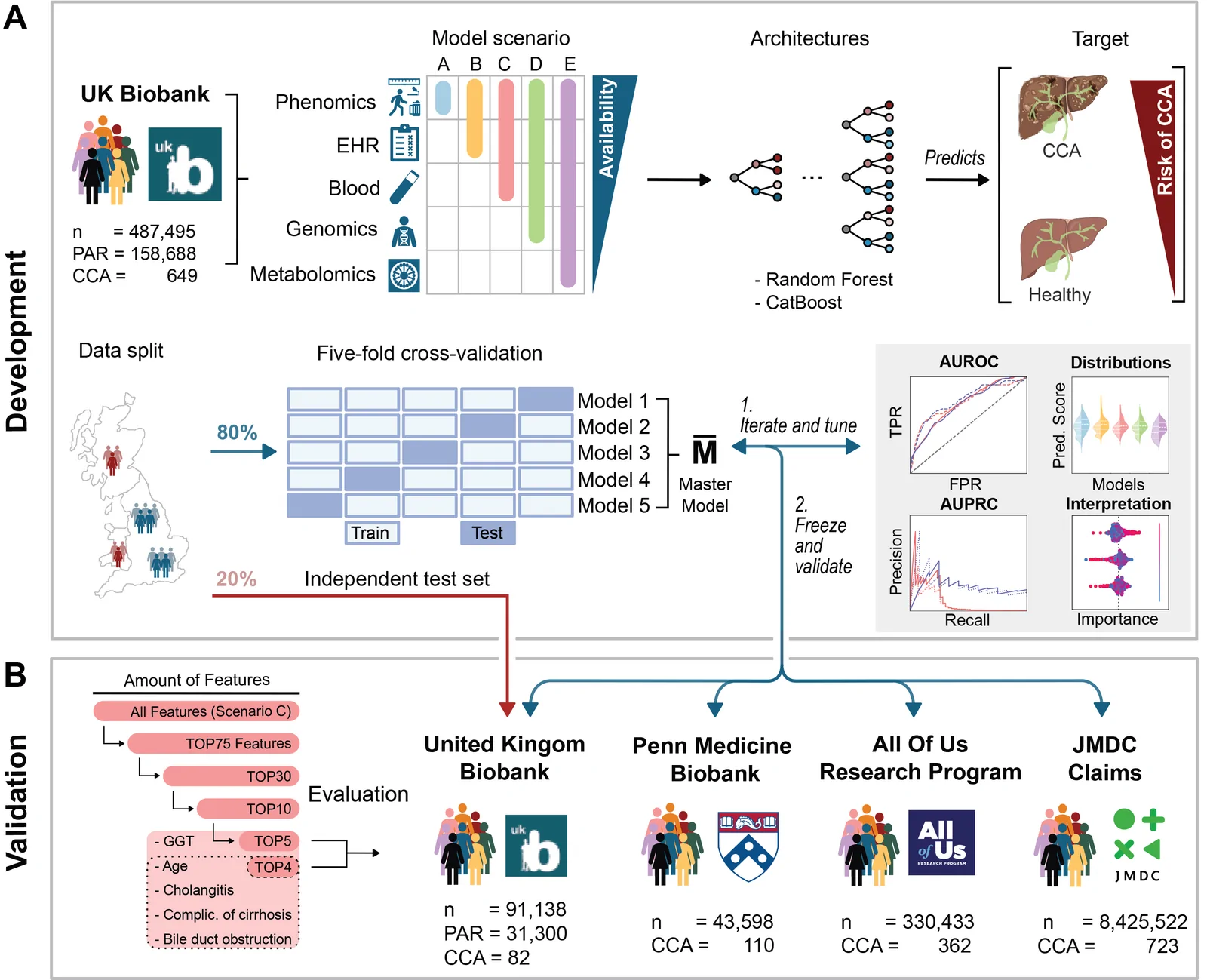
Study overview. (A) Overview of the study cohort, including all participants (n = 487,495), identified cholangiocarcinoma (CCA) cases (n = 649), and a subset of patients at risk (PAR cohort, n = 158,688) in the UK Biobank. Five scenarios (A–E), defined by the availability of different data modalities, were used to train Random Forest (RFC) and CatBoost models to predict risk of future CCA diagnosis. Models were trained and tested on 80% of the data (from English assessment centres) using five-fold cross-validation, then combined into a master (mean-voting) model, with reiteration and tuning after performance evaluation. AUROC: Area Under Receiver Operating Characteristics curve; AUPRC: Area Under Precision Recall Curve. (B) Validation of the trained models after feature ablation based on “feature importance” and “feature availability” to derive a minimal, widely applicable model. Independent performance evaluation was conducted on the withheld 20% of UKB participants from Wales, Scotland, and Newcastle (n = 91,138), as well as externally validated in the Penn Medicine Biobank (PMBB, n = 43,598), the All of Us Research Program (AOU, n = 330,433), and JMDC Claims Database (n = 8,425,522) for the TOP4 and TOP5 model.

Large prospective resources such as the UK Biobank (UKB) and the All of Us Research Program (AOU), alongside health-system biobanks like the Penn Medicine Biobank (PMBB) and federated electronic health record (EHR) networks like the Japan Medical Data Centre Claims Database (JMDC) or TriNetX offer extensive multimodal data and represent ideal resources to develop population-based risk stratification tools.^31^, ^32^, ^33^ Here, we developed and tested machine learning models that predict future CCA risk on multimodal population-based data from the UKB (Fig. 1A, Supplementary Tables S1–S12) and externally validated the final five-parameter score in PMBB, AOU, and JMDC, with exploratory feature-based application in TriNetX (Fig. 1B).

## Methods

### The UK biobank cohort

The UKB (RRID:SCR_012815) is a prospective cohort study that enroled 502,411 participants aged 37–73 from 22 centres across Scotland, Wales, and England between 2006 and 2010. Participants provided written consent for genetic analysis and longitudinal linkage to EHR, including hospital admissions, cancer registry entries, and death records. As of January 2026, data from 501,941 participant records remained available, following the withdrawal of consent of 470 individuals. The UKB received ethical approval from the Northwest Multi-Centre Research Ethics Committee. Additional details are available on the UKB website.^34^

### Baseline assessment in UKB

Participants completed touchscreen questionnaires capturing demographic information, lifestyle factors, and current medications. Participant sex (Data-Field 31) was derived from National Health Service (NHS) central registry records at recruitment and updated upon self-report if discordant. Recruitment planning aimed for an approximately equal distribution of male and female participants. In the demographic analysis, data are reported aggregated by sex, and sex was subsequently included as an independent variable in the development of CCA.^35^ A comprehensive list of all baseline measurements included in this study is provided in Supplementary Table S1. Urine and blood samples (see *Blood data*) were simultaneously collected at baseline assessment for subsequent genetic and metabolomic analyses.

### Electronic health records

EHR data from the initial UKB baseline assessment were supplemented with records from subsequent visits to the National Health Service (NHS) through International Classification of Diseases (ICD)-based mapping of primary and secondary diagnoses (Supplementary Tables S2–S4). Additionally, self-reported conditions were incorporated. EHR data were supplemented with data from the respective national death and cancer registries. We further conducted a phenome-wide association study (PheWAS) to identify clinical diagnoses associated with CCA status. ICD codes were mapped to phecodes and associations were tested using logistic regression adjusted for age, sex, body mass index (BMI), and socioeconomic status (Townsend index).

### Cohort definition

**Fig. 2:**
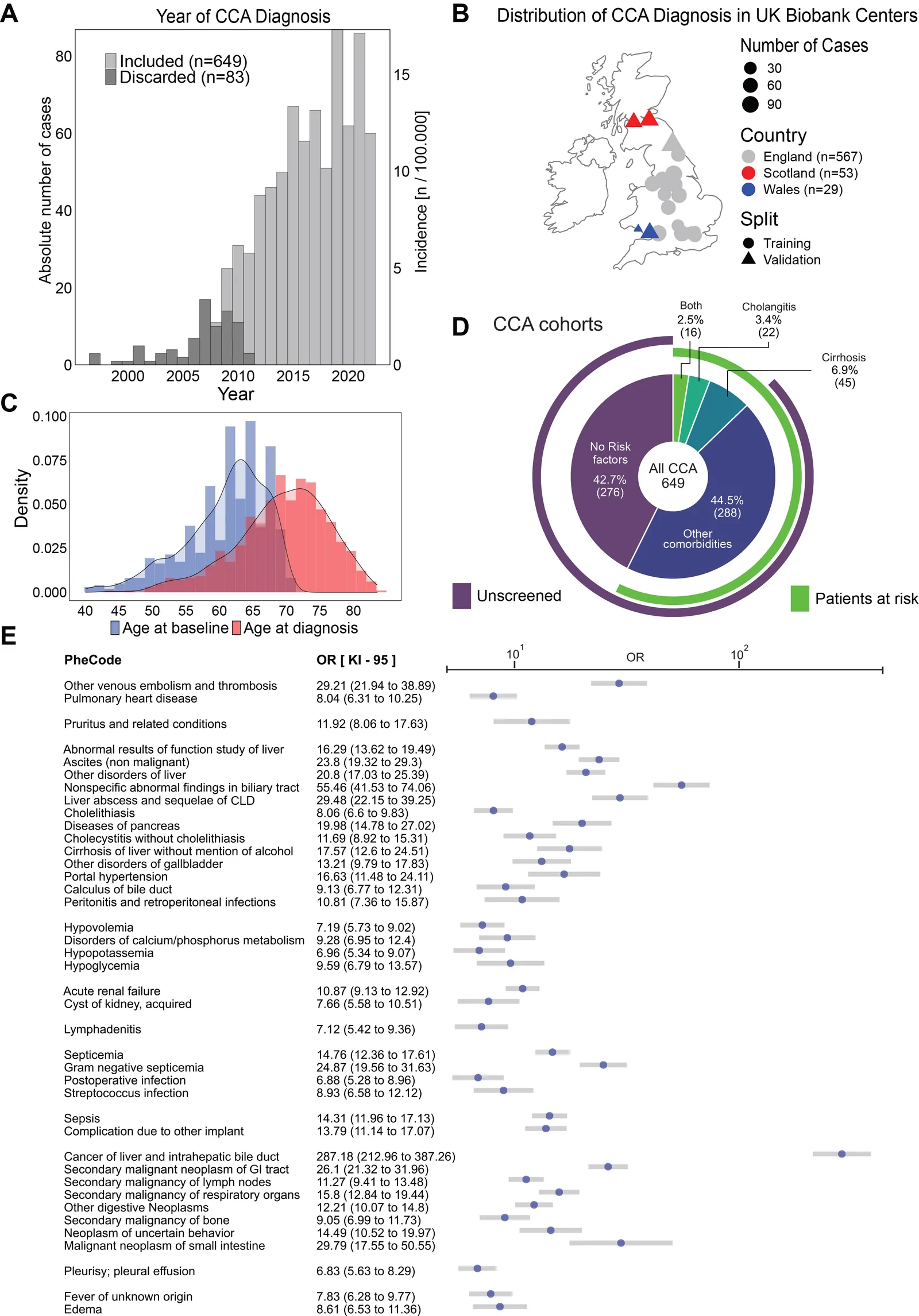
Clinical and Demographic Characterisation of Cholangiocarcinoma (CCA) Cases in the UK Biobank. (A) Histogram showing included (light grey) and excluded (dark grey) CCA cases by year of initial EHR diagnosis. (B) Map Plot of the United Kingdom, with cases and assigned split for each country included in the UK Biobank. (C) Age density histogram with age at baseline assessment and at diagnosis of CCA; Density represents relative amount of patients in specific age bins. (D) Donut plot for cohort distribution based on comorbidities of patients with CCA; Cholangitis and Cirrhosis ICD-Codes as well as Patients at risk (PAR) inclusion criteria are disclosed in Supplementary Tables S15 and S16. Both: Cholangitis and Cirrhosis. (E) Forest plot of Phenome wide association study for all CCA vs. control group; adjusted for age, sex, BMI and Townsend index; logarithmic Odds ratio (OR) depicted by blue dots, with 95% confidence intervals (CI) depicted by grey bars; Phecodes on the left are grouped by PheCode group (bold); Cancer of liver and intrahepatic bile duct depicts other, non-CCA liver cancers, with CCA cases removed before analysis; CLD: Chronic liver disease; GI: gastrointestinal.

Participants with relevant ICD-10 codes identified in hospital EHRs, cancer registries, or death registries were classified as CCA cases (CCA-All): C22.1, C24.0, C24.1, C24.8, or C24.9. Individuals with CCA-related entries recorded before or within one year of baseline assessment were excluded (Fig. 2A). Additionally, individuals with ICD-code of pancreatic cancer were excluded from analysis.

### CCA subtype definition

Subtype definitions for intrahepatic cholangiocarcinoma (iCCA) and extrahepatic cholangiocarcinoma (eCCA) were based on ICD-10 codes recorded in EHR and cancer registry data, with iCCA defined by code C22.1, while eCCA included codes C24.0, C24.1, C24.8, and C24.9 (see cohort definition). Participants with overlapping diagnoses of both subtypes were excluded from the respective subtype-specific cohorts.

### Blood data

UKB blood assays were derived from EDTA blood samples analysed immediately after collection. All included features, units, and amounts of missingness are listed in Supplementary Tables S5 and S6. Oestradiol and Rheumatoid factor were excluded from analysis due to high amounts of missing values (402,879 and 437,202, respectively). Participants with >80% missing values were excluded from all subanalyses (12,181 participants). Elevated liver enzymes were defined as any value exceeding sex-specific thresholds (female/male): Aspartate aminotransferase (AST; 35/50 U/L), alanine aminotransferase (ALT; 35/50 U/L), gamma glutamyl transferase (GGT; 40/60 U/L), and alkaline phosphatase (AP; 105/130 U/L). This composite feature was used solely for cohort inclusion and was not included in model training.

### Genetics

Genetic data were available for a subset of 487,495 participants. Based on an extensive literature review,^36^^,^^37^ we identified CCA-associated genetic risk variants (single nucleotide polymorphisms; SNPs) (Supplementary Table S7). Genotype data were retrieved from UKB using command-line utility *gfetch* and processed with *PLINK* for extraction of variants of interest, alignment, and quality control. Participants were stratified by genotype into non-carriers, heterozygous carriers, and homozygous carriers of the risk allele.

### Metabolomics

Frozen EDTA plasma samples from 501,935 participants were analysed using high-throughput nuclear magnetic resonance (NMR) spectroscopy (Category 220) to quantify 249 metabolomic and lipidomic markers, with 168 absolute concentrations and 81 concentration ratios. All included features, units, and amounts of missingness are listed in Supplementary Tables S8 and S9. Participants with 100% missing values in the metabolomics data were excluded from all subanalyses based on metabolomics, leaving 488,082 participants for model training and testing. In combination with the removed cases in the blood data (see *Blood Data*), 482,322 cases with complete blood and metabolomics data were included in analyses.

### Data preprocessing

Data extraction and preprocessing were conducted via R and RStudio (R version 4.4.2 (2024-10-31); RStudio version 2024.12.0 + 467). Summary tables were created using the gtsummary package.^38^ Field IDs of all features used in this study are available in the Supplement (Supplementary Tables S1–S9). Categorical variables were treated as independent factor levels. Participants were stratified into six groups based on their assessment centres. Groups 1 to 5 were used for five-fold cross-validation (training and testing), while group 6 served as an independent test set for performance assessment (Fig. 2B, Supplementary Tables S10 and S11). Model training was performed on centres in England, approximating 80% of the total UKB population, whereas performance assessment used data from Wales, Scotland, and Newcastle (England) to approximate 20% of the total UKB population. Missing values in continuous variables were handled using a multivariate imputation by chained equations (MICE) approach, in which numeric variables with missingness were iteratively imputed from other numeric predictors (Supplementary Table S12). Imputed values were constrained to only include non-negative values to ensure physiological plausibility. Imputation was only performed on training data.

### Modelling and hyperparameter tuning

Modelling was performed using scikit-learn (version 1.2.2) and CatBoost (version 1.2.7).^39^^,^^40^ We applied a grid search for each estimator, with predefined hyperparameter options and final configurations for each model and estimator shown in the Supplementary Tables S13 and S14. Training was conducted using five-fold cross-validation on the stratified subgroups described in *Data preprocessing*. Cross-validated models were aggregated into a mean-voting ensemble and evaluated on the independent cohorts.

### Statistics

Baseline characteristics between cases and controls were compared by applying Welch’s two-sample t-test to continuous variables and χ^2^ tests to feature frequencies of categorical variables, as well as Benjamini and Hochberg or Bonferroni corrections for multiple testing. Model performance was evaluated using the Area Under the Receiver Operating Characteristic Curve (AUROC) values, Area under the Precision-Recall Curve (AUPRC) values, feature importance, and SHapley Additive exPlanations (SHAP) analyses. The AUROC and AUPRC values reported in this study represent mean performance metrics computed across the full observation period. AUROCs were compared using DeLong tests with Bonferroni corrected p-values (Supplementary Tables S17–S21). Thresholds were selected based on Youden’s J index.^41^ In addition, we examined balanced accuracy and the F-β score (β = 10) to identify clinically meaningful operating points with increased weight on sensitivity.^42^ All statistical metrics for every model used are summarised in Supplementary Tables S22–S25.

### Feature selection

Feature importance was calculated using scikit-learn’s impurity-based method, which quantifies each feature’s contribution to reducing impurity at each split across all trees, with higher values indicating greater impurity reduction.^39^ SHAP explains the output of the models by assigning an importance value to each feature for a particular prediction.^43^^,^^44^ Feature ablation was conducted by manual selection for clinical availability, direct reduction to top k features (75, 30, 20, 10, 5, 4) as well as by stepwise (n-1) reduction (Supplementary Table S26).

### Time-dependent sensitivity analysis

We performed a time-dependent performance analysis by redefining the prediction target to include only cases diagnosed within predefined time intervals after baseline (e.g., 3, 5, 6, 7, 8, 9, and 10 years). At each time horizon, model performance was evaluated on the full cohort using AUROC and AUPRC based on the predicted risk scores (Supplementary Table S27). 95% CI intervals were estimated using non-parametric bootstrap resampling of individuals with replacement (1000 iterations per time horizon).

### Model calibration and clinical utility

Calibration was performed post hoc on the UK Biobank training set using internal cross-validation. We applied Platt scaling (sigmoid calibration), implemented via scikit-learn’s CalibratedClassifierCV with 5-fold cross validation across assessment centres, as per our training protocol. Calibration quality was quantified using calibration intercept, calibration slope, and overall calibration error using the Brier score and log loss. Visual assessment was based on decile-grouped calibration plots (mean predicted risk vs. observed event rate) and distributional comparisons of predicted risks before and after calibration. The calibrated models were then deployed without refitting to the UKB holdout set and to external validation cohorts.

Clinical utility was evaluated via Decision Curve Analysis (DCA) to quantify net benefit across a range of decision thresholds. We report the standardised net benefit (sNB).^45^^,^^46^ Uncalibrated and calibrated model curves were evaluated against default strategies of universal intervention (“treat all”) and non-intervention (“treat none”). This analysis was used to identify a plausible threshold window for clinical evaluation across the validation cohorts.

### The Penn Medicine Biobank

The PMBB (RRID:SCR_022415) is an academic biobank that has enroled over 260,000 patients to date receiving clinical care within the University of Pennsylvania Health system. We accessed data for 43,598 participants with available blood samples, of whom 110 had a recorded CCA diagnosis by 2022. For the final model, 2638 patients were included in the analysis. All participants provided informed consent as part of PMBB enrolment, allowing access to their EHR data, laboratory values, and whole-exome sequencing data provided by Regeneron Genetics Centre. We extracted ICD-10 diagnoses and median clinical laboratory results directly from the Penn Health System EHR. Due to high missingness (up to 94% in GGT), we restricted analyses including GGT only to participants with complete data, except for pack-years, where missing values were imputed as zero. Models that did not include GGT were evaluated on the full cohort. PMBB data were converted to UKB units and normalised according to the UKB preprocessing (see Blood data) (Supplementary Tables S28 and S29). Further information on the PMBB can be found on the biobank’s official website.^47^

### The All of Us Research Program

The AOU cohort (RRID:SCR_027032) is a longitudinal cohort currently encompassing >865,000 participants as of Aug 2025.^48^ Health data are gathered through healthcare system visits and participant surveys, with more information available on their website.^49^^,^^50^ AOU combines data from >50 healthcare providers and harmonises it to the Observational Medical Outcomes Partnership (OMOP) common data model accessible to researchers. A total of 330,433 observations from 167,782 participants with available baseline demographic, lifestyle questionnaire, and blood data were included in the validation analysis. Preprocessing steps were performed in line with UKB preprocessing, however single data points were aggregated per year and validation was performed prospectively on the five-year period following assessment. Missing values were imputed within AOU using multiple imputation by chained equations (MICE) with Classification and Regression Trees (CART). Rows with remaining missing values after MICE were dropped (21%). AOU data were converted to UKB units according to the documentation in Supplementary Tables S30 and S31. Detailed cohort information can be found in Fig. 2 and Supplementary Tables S32 and S33.

### TriNetX

The data used in this study were collected on July 21, 2025 from the TriNetX Research Network (RRID:SCR_022760), providing access to real-time EHR (diagnoses, procedures, medications, laboratory values, genomic information) from approximately 155 million patients from 104 healthcare organisations.^33^ Owing to the aggregate-level nature of the TriNetX analytics environment, patient-level discrimination metrics such as AUROC and AUPRC cannot be computed. Consequently, these analyses were not meant as a full external validation, but rather as a contextual assessment of group-level risk associations with the features included in our prediction score. We compared five groups based on the TOP5 model (excluding age due to platform constraints): [1] a healthy control group; [2] individuals with elevated GGT (cut-offs detailed in *Blood data*); [3] individuals with any single TOP5 feature excluding GGT; [4] individuals with elevated GGT and at least one additional TOP5 feature; and [5] individuals with all TOP5 features. The healthy group included individuals with at least one documented blood pressure measurement and no recorded evidence (i.e., no positive entry) of any TOP5 features or any other established CCA risk factors (Supplementary Table S34). Overlap with pancreatic cancer was excluded, consistent with UKB methodology. For group comparisons, TriNetX’s built-in propensity score matching was applied using age, BMI, and diabetes status as covariates (Supplementary Table S35). Within the matched cohorts, hazard and odds ratios for CCA were estimated using the aggregate-level time-to-event and outcome analysis tools provided by the TriNetX analytics environment. Analyses were stratified by sex, with separate evaluations for male and female participants.

### The JMDC

The JMDC database is a longitudinal administrative claims database with a total of 17,591,720 individuals cumulative participants registered between 2005 and 2023.^51^^,^^52^ Health data are gathered from multiple health insurance societies representing employees of Japanese corporations, integrating inpatient and outpatient medical claims alongside standardised annual health checkup results. Among these, 8,425,522 individuals who had available health checkup data and no prior history of cholangiocarcinoma (CCA) were included in the present analysis (Supplementary Table S36). Missing values within the JMDC data were imputed using multiple imputation by chained equations (MICE). Data source details have been described previously.^51^

### Code availability

All code developed and used throughout this study has been made open source and is available on GitHub at https://github.com/schneiderlabac/PRE_SCREEN_CCA.

### Role of funders

This study was supported by the German Cancer Aid (grant #70115730) and the Junior Principal Investigator Fellowship programme of RWTH Aachen Excellence strategy. The funding sources had no role in the study design; data collection, analysis, or interpretation; writing of the manuscript; or the decision to submit the manuscript for publication. The authors were not paid by a pharmaceutical company or any other agency to write this article. The corresponding author confirms that the authors were not precluded from accessing the data in the study and accepts responsibility for the decision to submit the manuscript for publication.

## Results

### Training cohort composition

649 participants with incident CCA and 487,495 overall UKB participants were included in the UKB development cohort “*All*”. 398 patients with CCA (61.3%) were confirmed by cancer registry records. Individuals with CCA-related EHR entries before or within one year of baseline assessment were excluded, to avoid training on features associated with present instead of future CCA (n = 83) (Supplementary Fig. S1, Fig. 1, Fig. 2A). Case distribution was homogeneous across assessment centres (Fig. 2B, Supplementary Tables S10 and S11). The mean time from initial assessment to diagnosis was 8.5 years and mean age at diagnosis was 69.9 ± 6.9 (Fig. 2C). The annual incidence rate in the UKB exceeded the average incidence per year in the UK (2.2/100,000), with a mean yearly incidence of 8.2/100,000.^53^

In addition to the *All* cohort, we established two additional cohorts (Fig. 2D). First, a *patients at risk (PAR)* cohort based on risk factors derived from literature and from our own Phenome Wide Association Study (PheWAS) (Fig. 2E).^8^^,^^17^, ^18^, ^19^ The *PAR* cohort included 158,688 individuals, with 366 positive CCA cases, with diagnosis of Chronic Liver Disease (CLD), cholelithiasis, cholangitis, elevated liver enzymes (AST, ALT, AP or GGT; see *Blood Data*) and other conditions associated with increased risk of CCA (Supplementary Table S15). Second, we derived a cohort to account for all patients that are not currently targeted by systematic screening procedures. Here, we excluded patients with pre-existing diagnoses of cirrhosis of any origin and cholangitis (Supplementary Table S16), as these diagnoses would justify screening for CCA as of current guidelines.^20^^,^^21^ This *Unscreened* cohort comprised 483,604 individuals with 566 positive CCA cases (87% of all CCA cases). Together, these data demonstrate that UKB provides a suitable training resource, combining a large incident CCA cohort with rich case numbers, comprehensive phenotypic characterisation, and risk factors aligned with those described in the literature.

### Test cohort compositions

To ensure robust development and independent validation, we trained our models on 80% of the UKB (England-based centres). The UKB test set, developed in a non-random split based on data from Scotland and Wales approximating 20% of the UKB population (Transparent reporting of a multivariable prediction model for individual prognosis or diagnosis, TRIPOD 2b^54^), included 91,138 participants with 82 CCA cases; of these, 31,300 participants met *PAR* criteria with 45 subsequent CCA cases and 90,471 participants met *Unscreened* criteria with 77 subsequent CCA cases. We then performed external validation in three independent cohorts without further subspecification of *PAR* or *Unscreened*: PMBB, AOU, and JMDC, as well as a linear derivative application of our model in the TriNetX cohort (TRIPOD 3).

Of the 43,598 available entries in PMBB, 2638 met the criteria for inclusion in the PMBB validation cohort for the TOP5 model, including 28 cases of CCA. Notably, the PMBB cohort comprised a significantly higher proportion of non-white participants compared to the UKB cohort (∼42% vs. ∼6%, Table 1, Supplementary Table S29).

**Table 1:** Baseline characteristics of UKB study population, stratified by sex and presence of CCA.

Characteristic	Female					Male				
	Overall N = 264,567^a^	CCA n = 329^a^	No CCA n = 264,238^a^	p-value^b^	q-value^c^	Overall N = 222,928^a^	CCA n = 320^a^	No CCA n = 222,608^a^	p-value^b^	q-value^c^
Age	56.4 (±8.0)	60.6 (±6.3)	56.3 (±8.0)	**<0.001**	<0.001	56.7 (±8.2)	61.2 (±6.0)	56.7 (±8.2)	**<0.001**	<0.001
Ethnicity				0.6	0.6				0.6	0.7
Unknown	1057 (0.4%)	<5	1057 (0.4%)			1243 (0.6%)	<5	1239 (0.6%)		
Caucasian	249,500 (94%)	318 (97%)	249,182 (94%)			209,728 (94%)	299 (93%)	209,429 (94%)		
Mixed	1778 (0.7%)	<5	1776 (0.7%)			1068 (0.5%)	<5	1068 (0.5%)		
Asian or Asian british	4398 (1.7%)	<5	4396 (1.7%)			5138 (2.3%)	7 (2.2%)	5131 (2.3%)		
Black or Black british	4401 (1.7%)	<5	4398 (1.7%)			3303 (1.5%)	6 (1.9%)	3297 (1.5%)		
Chinese	944 (0.4%)	<5	943 (0.4%)			562 (0.3%)	<5	561 (0.3%)		
Other	2489 (0.9%)	<5	2486 (0.9%)			1886 (0.8%)	<5	1883 (0.8%)		
BMI	27.1 (±5.2)	28.4 (±5.9)	27.1 (±5.2)	**<0.001**	<0.001	27.8 (±4.2)	28.7 (±4.7)	27.8 (±4.2)	**<0.001**	0.001
Waist circumference	84.7 (±12.5)	88.8 (±13.3)	84.7 (±12.5)	**<0.001**	<0.001	96.9 (±11.3)	100.5 (±13.0)	96.9 (±11.3)	**<0.001**	<0.001
Weight [kg]	71.4 (±14.0)	74.7 (±16.4)	71.4 (±14.0)	**<0.001**	<0.001	85.9 (±14.3)	88.4 (±16.3)	85.9 (±14.3)	**0.006**	0.009
Standing height	162.5 (±6.3)	162.2 (±6.6)	162.5 (±6.3)	0.4	0.6	175.6 (±6.8)	175.2 (±7.2)	175.6 (±6.8)	0.3	0.4
Multiple deprivation index	16.9 (±13.6)	17.1 (±13.4)	16.9 (±13.6)	0.8	0.8	17.5 (±14.2)	20.3 (±15.4)	17.5 (±14.2)	**0.001**	0.003
Bloodpressure sys. [mmHg]	135.3 (±18.7)	139.5 (±19.6)	135.3 (±18.7)	**<0.001**	<0.001	140.6 (±17.1)	144.9 (±17.5)	140.6 (±17.1)	**<0.001**	<0.001
Medication				**<0.001**	<0.001				**<0.001**	<0.001
No Medication	182,272 (69%)	200 (61%)	182,072 (69%)			149,172 (67%)	166 (52%)	149,006 (67%)		
Metabolic	61,153 (23%)	111 (34%)	61,042 (23%)			73,756 (33%)	154 (48%)	73,602 (33%)		
Hormones	21,142 (8.0%)	18 (5.5%)	21,124 (8.0%)			<5	<5	<5		
Family diabetes				0.5	0.6				>0.9	>0.9
0	213,437 (81%)	260 (79%)	213,177 (81%)			182,360 (82%)	263 (82%)	182,097 (82%)		
1	51,130 (19%)	69 (21%)	51,061 (19%)			40,568 (18%)	57 (18%)	40,511 (18%)		
Smoking status				**<0.001**	0.001				**0.003**	0.006
Never	156,969 (59%)	165 (50%)	156,804 (59%)			108,742 (49%)	123 (38%)	108,619 (49%)		
Previous	102,385 (39%)	161 (49%)	102,224 (39%)			106,261 (48%)	184 (58%)	106,077 (48%)		
Current	3956 (1.5%)	<5	3955 (1.5%)			6783 (3.0%)	11 (3.4%)	6772 (3.0%)		
Unknown	1257 (0.5%)	<5	1255 (0.5%)			1142 (0.5%)	<5	1140 (0.5%)		
Ever smoked				0.072	0.11				0.15	0.2
0	117,913 (45%)	126 (38%)	117,787 (45%)			77,020 (35%)	94 (29%)	76,926 (35%)		
1	145,369 (55%)	201 (61%)	145,168 (55%)			144,757 (65%)	224 (70%)	144,533 (65%)		
Unknown	1285 (0.5%)	<5	1283 (0.5%)			1151 (0.5%)	<5	1149 (0.5%)		
Pack years	6.5 (±11.7)	11.4 (±16.9)	6.5 (±11.7)	**<0.001**	<0.001	10.4 (±16.7)	15.1 (±21.0)	10.4 (±16.7)	**<0.001**	<0.001
Alcohol (gram per day)	6.6 (±8.7)	6.2 (±9.0)	6.6 (±8.7)	0.5	0.6	14.5 (±15.1)	14.9 (±16.4)	14.5 (±15.1)	0.7	0.7

Baseline characteristics of participants with available genotype data (N = 487,495) stratified by sex and CCA status (n = 649). Values are presented as mean ± standard deviation (SD) for continuous variables, and number (%) for categorical variables. Statistical significance was assessed using Welch’s two-sided t-test for continuous variables and chi-square test for categorical variables, with Benjamini and Hochberg-adjusted p-values (q-values) correcting for multiple comparisons, with p-values < 0.05 (statistically significant) depicted in bold.

a Mean (±SD); n (%).

b Welch Two Sample t-test; Pearson’s Chi-squared test.

c Benjamini & Hochberg correction for multiple testing.

The AOU cohort comprised 330,433 instances with 362 CCA cases from 167,782 unique participants. Ethnicity distribution was more consistent with the general US ethnicity distribution. Specific cohort characteristics are listed in Table 2 and Supplementary Tables S32 and S33. The JMDC cohort included 8,425,522 patients with 723 CCA cases. In the TriNetX cohort, participants were stratified into predefined groups reflecting combinations of TOP5 model features, as described in the Methods section. Cohort sizes ranged from 2541 to 728,886 participants, with CCA cases ranging from ≤10 to 1592 (counts ≤10 are masked per TriNetX policy) (Supplementary Table S34). Together, these datasets enable two consecutive steps of external validation, providing a rigorous foundation to examine robustness and generalisability across distinct populations.

**Table 2:** Baseline characteristics of AOU study population, stratified by sex and presence of CCA.

Characteristic	Female					Male				
	Overall N = 200,184^a^	CCA n = 167^a^	No CCA n = 200,017^a^	p-value^b^	q-value^c^	Overall N = 130,249^a^	CCA n = 195^a^	No CCA n = 130,054^a^	p-value^b^	q-value^c^
Age [years]	56.7 (±15.5)	65.0 (±11.8)	56.7 (±15.5)	**<0.001**	<0.001	62.2 (±14.2)	66.2 (±12.1)	62.2 (±14.3)	**<0.001**	<0.001
BMI	31.5 (±8.3)	30.6 (±9.0)	31.5 (±8.3)	0.2	>0.9	30.1 (±6.6)	29.0 (±5.8)	30.1 (±6.6)	**0.006**	0.074
Waist circumference [cm]	97.6 (±18.0)	96.2 (±18.6)	97.6 (±18.0)	0.4	>0.9	104.7 (±16.2)	104.0 (±13.1)	104.7 (±16.2)	0.5	>0.9
Weight [kg]	82.9 (±23.1)	79.6 (±23.3)	82.9 (±23.1)	0.076	>0.9	93.3 (±22.0)	88.0 (±18.9)	93.3 (±22.0)	**<0.001**	0.002
Standing height [cm]	162.0 (±7.1)	161.9 (±6.8)	162.0 (±7.1)	0.8	>0.9	175.8 (±7.7)	174.3 (±7.2)	175.8 (±7.7)	**0.004**	0.057
Self-reported ethnicity				0.5	>0.9				0.9	>0.9
Asian	3593 (1.8%)	<20	3590 (1.8%)			2538 (1.9%)	<20	2533 (1.9%)		
Black/ African American	38,425 (19%)	37 (22%)	38,388 (19%)			19,229 (15%)	26 (13%)	19,203 (15%)		
Latinx	34,861 (17%)	24 (14%)	34,837 (17%)			15,727 (12%)	24 (12%)	15,703 (12%)		
Middle Eastern	731 (0.4%)	<20	731 (0.4%)			712 (0.5%)	<20	712 (0.5%)		
More than one	2820 (1.4%)	<20	2817 (1.4%)			1391 (1.1%)	<20	1389 (1.1%)		
No answer	5680 (2.8%)	<20	5671 (2.8%)			4360 (3.3%)	<20	4356 (3.3%)		
Pacific Islander	152 (<0.1%)	<20	152 (<0.1%)			117 (<0.1%)	<20	117 (<0.1%)		
White	113,922 (57%)	91 (54%)	113,831 (57%)			86,175 (66%)	134 (69%)	86,041 (66%)		
Multiple deprivation index	0.3 (±0.1)	0.3 (±0.1)	0.3 (±0.1)	0.088	>0.9	0.3 (±0.1)	0.3 (±0.1)	0.3 (±0.1)	0.7	>0.9
Bloodpressure sys. [mmHg]	126.7 (±17.8)	129.3 (±19.1)	126.7 (±17.8)	0.078	>0.9	131.0 (±16.8)	130.0 (±16.3)	131.0 (±16.8)	0.4	>0.9
Medication				0.8	>0.9				**0.042**	0.5
Hormones	2218 (1.1%)	<20	2217 (1.1%)			983 (0.8%)	<20	982 (0.8%)		
Metabolic	53,079 (27%)	44 (26%)	53,035 (27%)			42,131 (32%)	47 (24%)	42,084 (32%)		
No medication	144,887 (72%)	122 (73%)	144,765 (72%)			87,135 (67%)	147 (75%)	86,988 (67%)		
Diabetes mellitus	70,352 (35%)	84 (50%)	70,268 (35%)	**<0.001**	<0.001	52,853 (41%)	99 (51%)	52,754 (41%)	**0.005**	0.061
Family diabetes	33,800 (17%)	35 (21%)	33,765 (17%)	0.2	>0.9	15,880 (12%)	<20	15,862 (12%)	0.2	>0.9
Pack years	5.3 (±12.4)	6.8 (±11.5)	5.3 (±12.4)	0.10	>0.9	9.4 (±17.9)	12.1 (±20.5)	9.4 (±17.9)	0.064	0.8
Alcohol [g/d]	3.4 (±7.3)	3.0 (±8.1)	3.4 (±7.3)	0.6	>0.9	6.2 (±11.5)	5.4 (±12.6)	6.2 (±11.5)	0.4	>0.9

Baseline characteristics of participants (N = 330,433) stratified by sex and CCA status (n = 362). Values are presented as mean ± standard deviation (SD) for continuous variables, and number (%) for categorical variables. Statistical significance was assessed using Welch’s two-sided t-test for continuous variables and chi-square test for categorical variables, with Benjamini and Hochberg-adjusted p-values (q-values) correcting for multiple comparisons, with p-values < 0.05 (statistically significant) depicted in bold.

a Mean (±SD); n (%).

b Welch Two Sample t-test; Pearson’s Chi-squared test.

c Benjamini & Hochberg correction for multiple testing.

### Machine learning enables CCA risk stratification in general and high-risk cohorts

We hypothesised that iteratively adding clinically accessible modalities would incrementally enhance performance. To test this, we developed five prediction models with increasing data layers, reflecting data availability and cost in routine clinical settings. Model A (demographic data), Model B (A + electronic health records), Model C (B + blood biomarkers), Model D (C + genetic factors), and Model E (D + metabolomics) (Fig. 1A). Models A-C were developed and validated on the full set of participants (n = 487,495), Model D on all participants for whom genotype data were available (n = 487,495), and Model E on all participants for whom blood, genotypic and metabolomic data were available (n = 482,322).

**Fig. 3:**
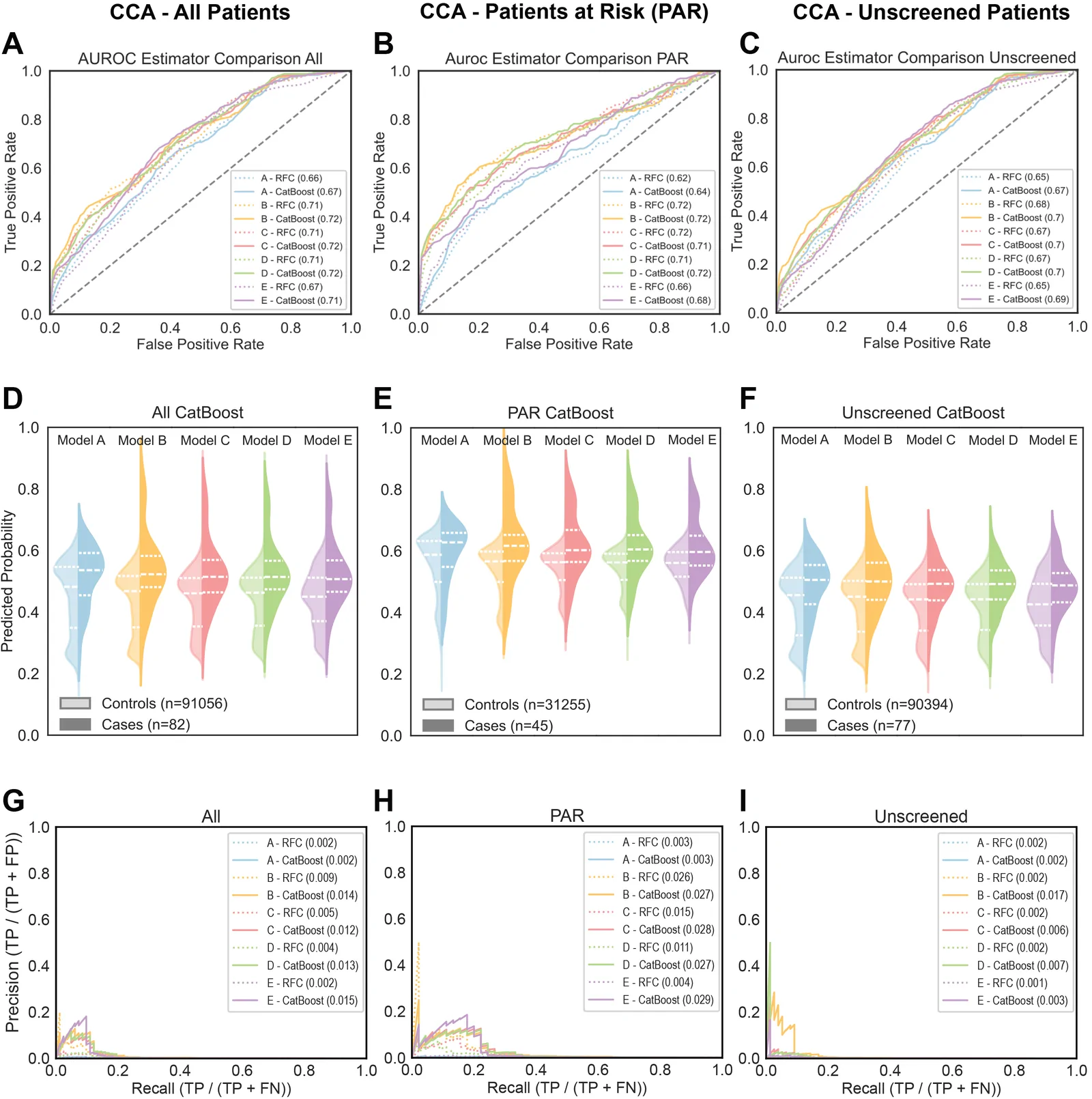
Model Performance All Cohort vs. Patients at Risk vs. Unscreened. Curves and plots shown for incremental Models A-E, respectively. (A)/ (B)/ (C) Receiver operating characteristics curves for CCA-All (A), CCA-Patients at Risk (PAR) (B) and CCA-Unscreened (C) for both Random Forest Classifier (RFC) and CatBoost; Area under the curve values depicted in the box for each curve; dashed line represents AUC of 0.5 (D)/ (E)/ (F) Violin plots for prediction values of cases and controls for CCA-All (D), CCA-PAR (E) and CCA-Unscreened (F) for the RFC Model; Dashed and dotted lines represent 50th, as well as 25th and 75th percentiles, respectively. (G)/ (H)/ (I) Precision Recall Curves for CCA-All (G), CCA-PAR (H) and CCA-Unscreened (I) for RFC and CatBoost; Zoomed in view in the middle; Area under the curve values depicted in the box for each curve. TP: True positives; FP: False positives; FN: False negatives.

In the CCA-All cohort, AUROC values in scenarios B-D were similar in both Random Forest Classifier (RFC) and CatBoost models, reaching a maximum of 0.72 [95%CI: 0.706–0.729] in Model C-CatBoost (Fig. 3A/D, Supplementary Table S22 and S24). CatBoost and RFC performed comparably, with CatBoost performing better in scenarios A and E (Supplementary Table S20). AUPRC values were generally low, in respect to the low prevalence of CCA in the general population, reaching around 0.002 [95% CI: 0.002–0.003]–0.015 [95% CI: 0.013–0.017], peaking in scenario E, with similar values for the CatBoost models in scenarios B-E (Fig. 3D/G).

Similar patterns were observed in the CCA-PAR cohort, where AUROC values peaked at 0.72 [95% CI: 0.712–0.728] (Model C-RFC, Fig. 3B, Supplementary Table S15). AUPRC values were slightly higher than in the CCA-All cohort, with 0.003 [95% CI: 0.003–0.003] in scenario A-CatBoost to 0.029 [95% CI: 0.025–0.040] in scenario E-CatBoost, but trends across estimators remained consistent (Fig. 3E/H). In the CCA-PAR cohort, RFC and CatBoost only showed significant differences in predictive performance for scenario A, with CatBoost performing slightly better (Supplementary Table S20).

### Subgroup analyses by risk profile and tumour type

Training models exclusively on the CCA-Unscreened cohort resulted in reduced performance compared to CCA-All or CCA-PAR. AUROC values ranged from 0.67 [95% CI: 0.658–0.676] (Model A) to 0.7 [95% CI: 0.694–0.705] (Model C) and 0.65 [95% CI: 0.647–0.661] (Model A) to 0.68 [95% CI: 0.676–0.689] (Model B) for CatBoost and RFC, respectively (Fig. 3C, Supplementary Table S19). AUPRC values were similar across models, with a maximum of 0.017 [95% CI: 0.012–0.024] for Model B-CatBoost (Fig. 3F/I). These shifts suggest that predictive performance in individuals not currently included in routine surveillance may follow different patterns compared to the *CCA-All* and *CCA-PAR* cohorts (Supplementary Fig. S3A–D for *CCA-Unscreened* and E-H for *CCA-PAR*). Although features reflecting advanced biliary or liver disease contributed to overall model performance, the model retained meaningful discriminative ability in individuals without prior screening indications, supporting its intended use as a population-level risk stratification and triage tool.

**Fig. 4:**
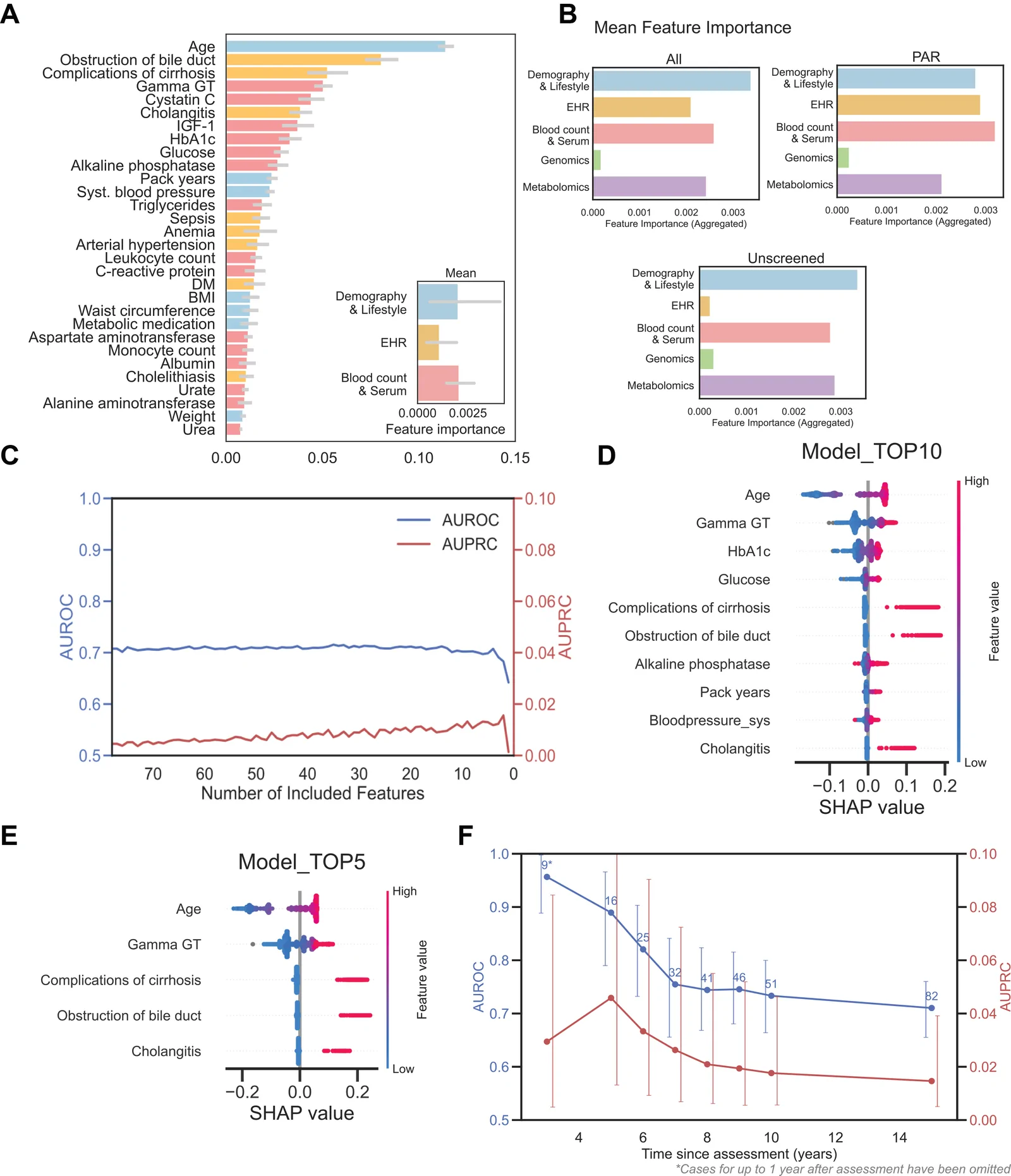
Model interpretability, feature reduction, and time-dependent evaluation. (A) Random Forest feature importance bar plot for CCA-All Model C, top 30 features ordered by importance for model prediction, bar colours depict group association with mean feature importance by group shown in lower-right box; positive 95% CI (grey bars) cut off for better readability. Gamma GT: Gamma Glutamyltransferase; IGF-1: Insulin-like Growth Factor 1; DM: Diabetes Mellitus; EHR: Electronic Health Records (B) Sum of RFC feature importance for model output ordered by feature group for Model B (top graph) and Model E (bottom graph) (C) Area Under Receiver Operating Characteristics curve (AUROC) (blue) and Area Under Precision Recall curve (AUPRC) (red) values for stepwise (n-1) feature reduction of 154 features in Random Forest Classifier Model C to 1 feature, based on feature importance (D/E) SHapley Additive exPlanation (SHAP) beeswarm plot of RFC models with top 10 and 5 features ordered by impact on model output; Colours indicate original feature value (blue = low, red = high), values left and right of the middle account for negative and positive impact on model output, respectively. (F) Time dependent readout of AUROC (blue) and AUPRC (red) values for Model TOP5 RFC; annotated on the AUROC curve are the number of cases available to the model at each timepoint (3, 5, 6, 7, 8, 9, 10 and full observation period) on the y-axis. ∗ Cases for up to one year after assessment have been omitted.

Notably, predictive performance in the *CCA-All* and *CCA-PAR* cohorts plateaued after the addition of EHR data in scenario B, with only modest gains in AUROC and AUPRC following the inclusion of blood parameters in scenario C (Fig. 3A/B/G/H; Supplementary Tables S17 and S18). The addition of genomic and metabolomic data (scenarios D and E) did not yield further improvements. No SNPs ranked among the top 30 most important features in any model (Fig. 4A/B), while metabolomics provided only a minor impact on model outcome (Fig. 4B). We therefore intentionally proceeded with Model C (demographics, EHR and blood) as our primary baseline for consecutive ablation studies, due to its reliance on exclusively routine clinical data and superior performance (Fig. 3A/B/C, Supplementary Fig. S4A and B, Supplementary Table S17).

Given the distinct risk factor profiles for iCCA and eCCA, we hypothesised that training separate models for each subtype would improve performance compared to a single, combined model. Exploratory sensitivity analyses stratified by intrahepatic and extrahepatic cholangiocarcinoma did not yield improved predictive performance compared with the unified *CCA-All* cohort and are reported in detail in the Supplementary Materials (Supplementary Fig. S2A–F).

### Random forest classifier identifies risk factors for CCA

Feature importance exploration via mean decrease of impurity and via SHAP revealed key predictors of CCA development. Age consistently ranked as the most important feature across all models (Fig. 4A, Supplementary Fig. S3). Other demographic and lifestyle factors included pack-years, systolic blood pressure and waist circumference, with significant differences between cases and controls presenting in cohort comparison (Fig. 4A and Table 1). Interestingly, while pack-years were positively correlated to the presence of CCA, alcohol consumption was not significantly different and was not present in any feature importance or SHAP analysis (Fig. 4A/D/E). Among EHR features, presence of obstruction of the bile duct, complications of cirrhosis and cholangitis (which includes PSC in ICD10-encoding) were influential on model output (Fig. 4D/E). Other correlating diagnoses included sepsis, arterial hypertension and *diabetes mellitus*, as well as cholelithiasis (Fig. 4A). In blood parameters, liver enzymes ranked high across all models, with GGT consistently ranking highest (Fig. 4A). Other blood values included diabetes associated parameters like haemoglobin A1c (HbA1c) or blood glucose as well as cystatin C, insulin-like growth factor 1 (IGF-1) and leucocyte count.

### Ablation studies show retained performance in slim models and better short-term performance

Feature reduction is critical for improving clinical applicability of predictive models. We aimed to reduce the number of input variables and minimise noise while maintaining predictive accuracy. We therefore performed a stepwise feature ablation analysis and reduced the number of features from 154 in Model C to 10 and finally only 5 features in the TOP10 and TOP5 RFC models. The TOP10 model included age, GGT, HbA1c, blood glucose, systolic blood pressure, pack years, and AP, as well as the presence of cholangitis, complications of cirrhosis, or obstruction of bile duct (Fig. 4D). The TOP5 model retained age, GGT and presence of cholangitis, obstruction of bile duct and complications of cirrhosis (Fig. 4E).

**Fig. 5:**
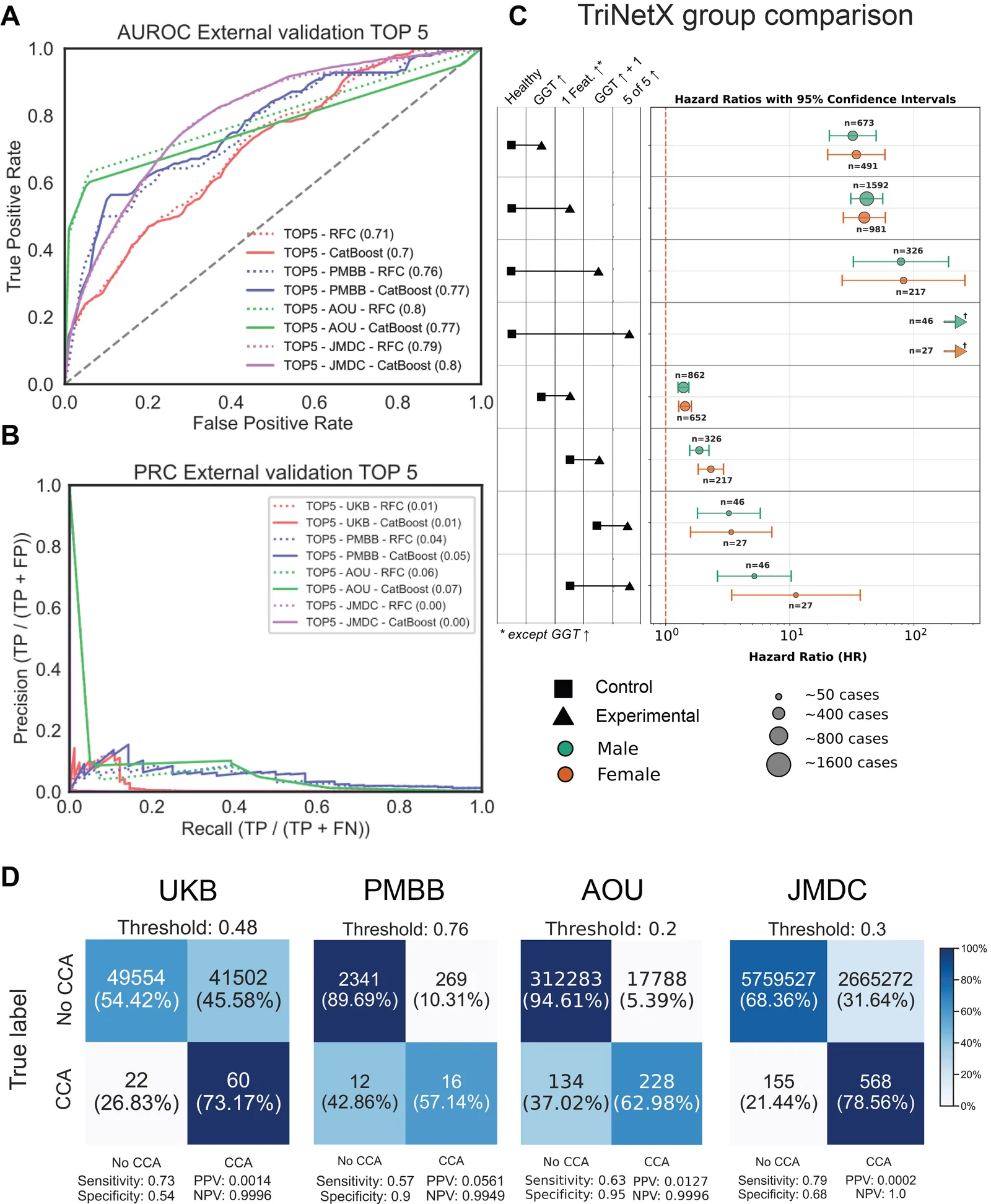
External validation in Penn Medicine Biobank, All of Us Research Program, JMDC and TriNetX. (A) Receiver operation characteristics curves for Model TOP5 for Random Forest Classifier (RFC, dotted) and CatBoost (line) in UKB (red), PMBB (blue), AOU (green) and JMDC (purple) cohorts, with area under the curve (AUC) values in the bottom right corner. Dashed grey line depicts AUC of 0.5 (B) Precision recall curves for Model TOP5 for RFC (dotted) and CatBoost (line) in UKB (red), PMBB (blue), AOU (green) and JMDC (purple) cohorts; AUC values in the upper right corner. TP: True Positives; FP: False Positives; FN: False Negatives. (C) On the left, control groups (rectangles) and experimental groups (triangles) are defined by inclusion criteria based on the TOP5 model (see *External Validation/TriNetX*). The forest plot on the right displays hazard ratios (HRs) for each comparison and 95% confidence intervals at each side; the y-axis is on a logarithmic scale. Sex is indicated by colour (male: turquoise; female: orange), and the size of each point reflects the number of CCA cases in the experimental group. † In the comparison between the healthy group and the “5 of 5” group, no matched cases remained in the control group, rendering HR calculation infeasible. GGT = Gamma glutamyltransferase, Feat. = Feature. (D) Confusion matrices for CatBoost Model TOP5 in UKB, PMBB, AOU and JMDC from left to right for different thresholds calculated through Youden J Index per cohort. PPV: Positive predictive value; NPV: Negative predictive value. Colouring indicates relative percentage per class.

Given the high proportion of missing GGT values in external validation cohorts, we additionally derived a TOP4 model excluding GGT to enable validation in our external cohorts without relying on imputation. This feature ablation of >90% of initial features mostly preserved model performance, with AUROC values of 0.71 [95% CI: 0.706–0.719 and 0.703–0.711, for TOP10 and TOP5 RFC respectively] and corresponding AUPRC values of 0.01 [95% CI: 0.010–0.017 and 0.009–0.018, respectively] (Fig. 4C and 5A/B, Supplementary Table S20). Further ablation to TOP4 reduced the AUROC to 0.69 [95% CI: 0.689–0.697] and the AUPRC to 0.015 [95% CI: 0.013–0.016]. CatBoost and RFC performed similarly in our analyses (Fig. 5A/B, Supplementary Fig. S4A and B, Supplementary Table S18). Notably, while the TOP5 and TOP10 models did not significantly differ in predictive performance, they were slightly, but significantly worse than the models with larger feature spaces (e.g., TOP75 vs. TOP5, adj.-p = 0.0003 (DeLong test); Supplementary Fig. S4A and B, Supplementary Table S21). The ablation resulted in a compact and clinically accessible feature set that retained most of the predictive signal and was therefore prioritised for external validation.

Given the long observation period of over 14 years in the UKB, we hypothesised that predictive performance would be highest in temporal proximity to initial assessment, particularly as blood samples were acquired only at baseline. Time-dependent analysis confirmed this hypothesis, with AUROC values in the UKB demonstrating a near perfect classification at year 3 0.96 [95% CI: 0.882–0.997] to 0.71 [95% CI:0.653–0.759] at year 14 (Fig. 4F, Supplementary Table S27). This pattern was consistent with the clinical reality that risk factors captured at baseline are most informative for near-term cancer development.^55^ Correspondingly, AUPRC values in the UKB exhibited a similar pattern, reaching their maximum of 0.046 [0.0113–0.1209] within the five-year prediction window, while all-time prediction AUPRC was 0.015 [95% CI: 0.0052–0.0364], (Fig. 4F). Together, these findings indicate that discrimination and precision are best within the first five years after assessment and decrease with increasing prediction horizons.

### Predictive models remain robust in external validation

Finally, we explored the generalisability of our models in the independent population-level cohorts PMBB (n = 43,598; 110 cases of CCA), AOU (n = 330,433; 362 cases of CCA) and JMDC (n = 8,425,522; 723 cases of CCA). While similar data to UKB was available in PMBB, AOU and JMDC, key differences encompassed a different ethnic background (Tables 1 and 2, Supplementary Table S29) and different methods of recruitment,^51^^,^^56^^,^^57^ with resulting differences in disease burden (Supplementary Tables S4 and S32). We chose to validate the following models: TOP5-CatBoost, TOP5-RFC, TOP4-CatBoost and TOP4-RFC.

In PMBB, our models achieved AUROC values of 0.77 and 0.832 [95% CI: 0.764–0.778 and 0.831–0.832, for CatBoost TOP5 and TOP4 respectively] with AUPRC values of 0.055 and 0.098 [95% CI: 0.054–0.056 and 0.091–0.106, CatBoost TOP5 and TOP4 respectively] (Fig. 5A/B, Supplementary Fig. S5A and B, Supplementary Tables S22 and S24). In AOU we achieved AUROC values of 0.796 [95% CI: 0.795–0.798 for RFC TOP5], with AUPRC values of 0.038 [95% CI: 0.033–0.042, for RFC TOP5] (Fig. 5A/B; Supplementary Table S5A and B).

As patient-level predictions were not possible in the TriNetX environment, we stratified patients in groups reflecting specific combinations of the models risk features. Both hazard ratios and odds ratios increased stepwise across these groups compared to the healthy reference group. The strongest associations were observed in individuals with multiple concurrent TOP5 features, with hazard ratios reaching up to 82.5 [95% CI: 26.4–257.96] in females and 78.7 [95% CI: 35.5–190.3] in males (Fig. 5C, Supplementary Fig. S6). Validation in JMDC, the largest epidemiological database from Japan, revealed AUROCs of 0.8 [95% CI: 0.794–0.805] and 0.794 [95% CI: 0.791–0.798] for TOP5-CatBoost and TOP5-RFC, respectively. AUPRC values were low with 0.001 [95% CI: 0.001–0.001] in both TOP5-CatBoost and TOP5-RFC (Supplementary Tables S22 and S24).

To facilitate clinical interpretation, we defined classification thresholds using Youden’s J Index (J = Sensitivity + Specificity − 1) within each cohort for our TOP5 model. At these thresholds, our models detected ∼ 73% of CCA cases (UKB), 57% (PMBB), 63% (AOU) and 79% (JMDC), corresponding to a number needed to screen of 692.7, 17.8, 79, and 4693.4, for UKB, PMBB, AOU and JMDC respectively (Fig. 5D). This translates to approximately 1.5 (UKB), 56.2 (PMBB), 12.6 (AOU) and 0.21 (JMDC) CCA cases identified per 1000 individuals screened (Fig. 5, Supplementary Figs. S5C and 7A/B, Supplementary Tables S20 and S22). Together, these results establish our models as feasible, scalable tools for early CCA detection with clinically meaningful performance.

Finally, calibration developed in the UK Biobank remained stable when applied to the AOU cohort (Supplementary Figs. S8A–E and S9A–E), with preserved discrimination and improved Brier score (Calibrated: 0.001) and log loss values (Calibrated: 0.009, Supplementary Table S37). Decision curve analyses of our models showed a positive standardised net benefit of up to 11%, most apparent in the 0.5–3% threshold range (Supplementary Fig. S10).

## Discussion

To address the unmet need for a population-level risk stratification tool for CCA, we developed and validated machine learning models that predict the risk of developing CCA. We explored a broad range of biomedical data modalities across four large-scale, prospective, population-level cohorts. By using demographic, EHR, and routinely measured laboratory features, our models achieve modest, but robust performance across several large-scale, diverse external datasets and the highest accuracy for CCA risk prediction models thus far. These findings support the feasibility of low-cost, scalable CCA risk prediction in routine care, offering a crucial step toward population-based prescreening and timely referral for diagnostic evaluation.

Prerequisites of risk stratification tools for clinical use cases include availability of data, predictive accuracy, robustness, fairness and transparency. We therefore chose a stepwise approach to explore the relevance of individual data modalities. We included ubiquitously available data first, supplementing EHR and blood data and gradually moving towards sophisticated omics data, assessing performance metrics for each. To ensure robustness and fairness, we evaluated the models resulting from this process on a broad range of datasets and across sub-populations (Fig. 3B/C). The model architectures RFC and CatBoost were included based on the following considerations: suitability for low-dimensional, tabular data, robustness against overfitting, computational efficiency as well as data efficiency, and, crucially, interpretability.^58^^,^^59^

AUROC values ranged from 0.66 to 0.72 in the internal test cohort, with the best-performing model consisting of demographic, EHR, and blood data. Estimator choice did not substantially influence overall performance. Adding EHR and blood data, respectively, improved both AUROC and AUPRC. Meanwhile the inclusion of genomic features did not affect performance. However, this might change once CCA-specific genome-wide association studies become available.^60^ Models with metabolomic features performed similarly to models without. Although metabolomic markers have been associated with predictive value in other cancers,^61^^,^^62^ their inclusion did not improve performance in our cohort. This may indicate that the metabolomic information available here was either not sufficiently disease-specific for CCA risk prediction or provides limited incremental value beyond the demographic, EHR, and routine blood-based predictors, potentially due to overlapping biological information. Given this limited additional value, we prioritised models without metabolomic and genomic features to increase clinical applicability and scalability. Sub-analyses for respective CCA subtypes iCCA and eCCA did not improve performance compared to the models predicting overall CCA. Similarly, individual model training on specific cohorts, namely *CCA-PAR* and *CCA-Unscreened*, did not meaningfully alter performance. Therefore, we focused consecutive analyses on the population-level *CCA-All* models for broadest utility.

Our analyses provide valuable insights into relevant risk factors, which predominantly align with risk factors reported in the literature.^8^^,^^17^^,^^18^ Most notably, this included age and GGT, highlighting both the sporadic age-dependent nature of CCA as well as the risk associated with cholestatic events and chronic biliary inflammation.^63^, ^64^, ^65^ Further, our models identified the metabolic markers waist circumference, BMI, blood glucose, and HbA1c as key predictors aligning with the role of hyperglycemia in CCA pathogenesis.^17^^,^^66^^,^^67^ Smoking-related variables, particularly pack-years, also ranked highly, supporting a positive association despite conflicting evidence in the literature.^68^, ^69^, ^70^, ^71^

Several features, like pancreatic and hepatocellular cancer, were excluded from training data to avoid potential data leakage. Bile duct obstruction however was retained as a predictor because based on prior studies and our PheWAS, which associated obstructive biliary disease occurring years before CCA as a sign of benign cholestasis.^17^ Since obstruction can also be a manifestation of established CCA, we mitigated reverse causation by excluding participants with CCA at baseline or diagnosed within 12 months of enrolment. This approach allowed to record this valuable information without conflating risk prediction with reverse causation from occult, already-present CCA.

Real-world applicability depends on clinical availability and cost of input features. We therefore actively reduced necessary input variables with the following strategies: First, we omitted several predictive features like low IGF-1 and high cystatin C due to low availability.^72^, ^73^, ^74^ Second, we iteratively removed overall low-ranking features in an ablation study. Here, we observed a consistent performance until reduction to the final 5 features, which informed the development and validation of the TOP5 model.

Model performance must consider the prediction horizon. Predictive accuracy was highest within the first 3–5 years after baseline, with a marked decline thereafter. This finding highlights the time window in which the model provides the most reliable risk stratification and supports its potential role in risk assessment during this period. Given that the score relies on readily available data, repeated assessment over time may be feasible in clinical practice, although this was not evaluated in the present study.

Finally, we validated our risk model in three large-scale independent external cohorts (PMBB, AOU and JMDC) as well as validation of a linear model of the included variables in TOP5 in the TriNetX cohort. Model performance in PMBB was comparable to UKB, and even exceeded performance of the UKB test set in both AOU and JMDC, even despite completely different ethnic composition across validation cohorts and compared to UKB.^31^^,^^32^ This consistency might reflect the closer temporal proximity of blood measurements to diagnosis in these cohorts, where AOU and JMDC performance reflects the high UKB 3–5 year performance discussed above, rather than all-time performance. The robust performance therefore overall supports the model’s applicability and generalisability across populations. To accommodate differences in prevalence and cases across the databases, we derived cohort-specific operating thresholds using the Youden J index.

Our study has several limitations. While our model establishes robust performance for CCA risk prediction, its performance does not reach that of our earlier work on HCC risk prediction.^75^ This phenomenon may reflect the presence of well-documented risk factors associated with HCC, or conversely, the relative absence of such factors in CCA. Furthermore, in HCC there are distinct precursory disease stages compared to CCA pathogenesis, influencing the model’s prediction. Nonetheless, our results are to be interpreted with caution, since, compared to other entities, CCA prediction remains challenging and model performance is modest.

Limited diagnostic granularity for cholangitis codes in UKB likely hinders representation of PSC, a key risk factor for CCA. Enhanced granularity in cholangitis aetiology and standardised documentation of associated risk factors, consistent with current guideline recommendations, could improve model performance.^20^^,^^21^

The extent of missing data, particularly in external validation cohorts, posed a substantial challenge. For example, GGT was missing in 94% of cases in PMBB and 97.5% in AOU. According to American College of Gastroenterology guidelines, GGT measurement is typically ordered only to confirm hepatic origin of an isolated alkaline phosphatase elevation, not routinely, and therefore its absence is not missing at random (NMAR).^76^ In our context, availability of GGT measurements was associated with clinical suspicion of biliary or hepatic disease. Compared to US-based cohorts, GGT is more routinely assessed in Europe^77^ as well as in UKB. We therefore opted for two separate strategies to mitigate this limitation: In PMBB, we excluded individuals without GGT measurements for the validation of our TOP5 model. This may introduce selection bias, since cases without suspected pathology are omitted. We therefore tested a second approach, imputing GGT, in the AOU and JMDC cohorts. This risks creating collider bias, as both the outcome (CCA) and the exposure (e.g., cholestatic liver disease) influence measurement likelihood, potentially skewing associations. However the consistent performance across both PMBB, AOU and JMDC suggests that our approach remains robust regardless of the applied imputation strategy. Given the high proportion of missing GGT values in external cohorts, we further derived a reduced TOP4 model excluding GGT to enable more reliable validation. This enabled us to use the whole PMBB cohort as validation, with AUROC values of up to 0.83 and 0.8 in the AOU cohort.

The low AUPRC and the positive predictive values (PPV) observed in our study reflect an inherent challenge when evaluating predictive performance for rare diseases such as CCA. Both metrics are strongly influenced by class imbalance and disease prevalence, and low values are therefore expected even for well-discriminating models. In this context, AUPRC should be interpreted with caution, as its value is largely affected by the underlying event rate.^78^ This is especially prominent when observing the AUPRC values in the JMDC external validation, due to the high class imbalance in this cohort (∼8 million controls to 723 cases). Importantly, our proposed model is not intended as a diagnostic test, but rather as a low-cost screening and triage tool to better allocate high-risk subgroups to further evaluation. In such screening contexts, metrics that quantify risk enrichment and screening efficiency are more informative than absolute PPV or AUPRC alone. In particular, the number needed to screen (NNS) provides a clinically meaningful measure of utility by quantifying how many individuals must be evaluated to identify one additional case.

Similarly, the moderate calibration classification stems from low event counts inherent to rare CCA prevalence, falling short of the 200-event external validation threshold recommended by Van Calster et al.^79^ However, the low Brier score and log loss suggest adequate overall agreement between predicted and observed risk at the population level, while Decision Curve Analysis indicated a small but positive net benefit across clinically plausible threshold probabilities, particularly between approximately 0.5% and 3.0%. These findings demonstrate that moderate calibration provides entirely adequate utility, fulfilling its established role as a practical and realistic benchmark for clinical decision support.

Despite these limitations, this study introduces a risk prediction algorithm to support the early identification of future CCA. The proposed model relies on GGT alongside features readily available through EHR, enabling a low-cost and broadly applicable strategy for identifying individuals at elevated risk. Owing to its simplicity and reliance on routinely collected information, the model is well suited for clinical integration as a first-line triage mechanism to guide targeted diagnostic evaluation.

To foster independent external validation, we release all source code used to develop our models. This resource could be used directly as a standalone screening tool or embedded into large language model (LLM)–based agents to support automated, context-aware clinical decision support.^58^ Together, this work represents an important initial step toward CCA screening and risk stratification, providing both a benchmark for future evaluations and a foundation for refinement as additional risk factors emerge.

## Contributors

FvH, JC and CVS conceived the plan for the analysis. FvH executed the preprocessing. PHK and JC built the preprocessing and modelling pipeline. FvH evaluated model performances. FvH wrote the manuscript. JC and CVS revised and corrected the manuscript. FvH and JC accessed and verified UKB data, provided by CVS and KMS. Data from the PMBB were provided by DR, with support by CVS. CVS and DR verified the PMBB data. RG performed analyses on the TriNetX cohort, with data provided by IZ. FvH and RG verified the TriNetX data. DYZ, JC, and IT performed the external validation of the models on AOU. IT and JC accessed and verified AOU data. SIW, TK and NT performed the external validation of the models on JMDC. SIW, TK, and NT accessed and verified the JMDC data. TS, NJ, AK, HYRH, PMR, JMB, AG, AS, JNK, and KMS provided invaluable feedback and insights on the project and manuscript.

All authors have read and approved of the final version of this manuscript.

## Data sharing statement

UK Biobank data, including NMR metabolomics, are publicly available to bona fide researchers upon application at http://www.ukbiobank.ac.uk/using-the-resource/. Detailed information on predictors and endpoints used in this study is presented in the Supplementary Materials. Penn Medicine Biobank data are accessible to authorised users via the University of Pennsylvania under https://www.itmat.upenn.edu/biobank/researchers.html. Data from the All of Us Research Program’s Controlled Tier Dataset v8 are available to authorised users on the Researcher Workbench. Data from the JMDC Claims Database are available to authorised users via the JMDC website. Data from the TriNetX research network are available to authorised users after application on their website.

## Declaration of interests

JC received honoraria from Johnson and Johnson. JNK holds shares in StratifAI, Synagen, Spira Labs, Tremont AI, and Saterra AI; is Co-PI on institutional research grants from GSK and AstraZeneca; participates on an Advisory Board for AstraZeneca, DoMore Diagnostics, Owkin and Panakeia and declares honoraria or consulting fees from AstraZeneca, Bayer, Bioptimus, Daiichi Sankyo, Eisai, Janssen, Merck, MultiplexDx, MSD, Novartis, BMS, Roche, and Pfizer. AS received honoraria as speaker from BMS, Roche, Servier, Ipsen, Lilly, AstraZeneca, MSD, Eisai, Taiho, Amgen, travel support from Ipsen, Servier, Pierre-Fabre, MSD, Eisai, AstraZeneca, and consulting fees from Eisai, MSD, Roche, Incyte, BMS. AG declares consulting services for Advanz, AstraZeneca, CAMP4 Therapeutics, Gilead, Ipsen, Signanthealth has received a research grant from Ipsen, travel support from Ipsen and Zydus. JMB received honoraria or consulting fees for Albireo-Ipsen, CIMABay, OWL-Rubió Metabolomics, Jazz, AstraZeneca, Servier, Mirum Incyte, Eisai, Advanz and Mega and received grants to his institution by Albireo-Ipsen, Incyte, Cymabay-Gilead, Taiho and Jazz with no relation to this manuscript. KMS declares funding for this project through the BMBF/MKW Excellence strategy, the NRW Rueckkehr Programme and DFG SFB 1382 A11 and B09 (Project-ID 403224013) and received honoraria or consulting fees for AstraZeneca, Falk, Abbvie and Ipsen with no relation to this manuscript. The authors have no further competing interest to declare.

## References

[1] Banales J.M., Marin J.J.G., Lamarca A. (2020). Cholangiocarcinoma 2020: the next horizon in mechanisms and management. Nat Rev Gastroenterol Hepatol.

[2] (2023). S-3 Leitlinie zur Diagnostik und Therapie des Hepatozellulären Karzinoms und biliärer Karzinome.

[3] Kendall T., Verheij J., Gaudio E. (2019). Anatomical, histomorphological and molecular classification of cholangiocarcinoma. Liver Int.

[4] DeOliveira M.L., Cunningham S.C., Cameron J.L. (2007). Cholangiocarcinoma: thirty-one-year experience with 564 patients at a single institution: Thirty-one-year experience with 564 patients at a single institution. Ann Surg.

[5] Nakeeb A., Pitt H.A., Sohn T.A. (1996). Cholangiocarcinoma. A spectrum of intrahepatic, perihilar, and distal tumors. Ann Surg.

[6] Banales J.M., Rodrigues P.M., Affò S. (2026). Cholangiocarcinoma 2026: status quo, unmet needs and priorities. Nat Rev Gastroenterol Hepatol.

[7] Khan S.A., Emadossadaty S., Ladep N.G. (2012). Rising trends in cholangiocarcinoma: is the ICD classification system misleading us?. J Hepatol.

[8] Khan S.A., Tavolari S., Brandi G. (2019). Cholangiocarcinoma: epidemiology and risk factors. Liver Int.

[9] Welzel T.M., McGlynn K.A., Hsing A.W., O’Brien T.R., Pfeiffer R.M. (2006). Impact of classification of hilar cholangiocarcinomas (Klatskin tumors) on the incidence of Intra- and extrahepatic cholangiocarcinoma in the United States. J Natl Cancer Inst.

[10] Bertuccio P., Malvezzi M., Carioli G. (2019). Global trends in mortality from intrahepatic and extrahepatic cholangiocarcinoma. J Hepatol.

[11] Saha S.K., Zhu A.X., Fuchs C.S., Brooks G.A. (2016). Forty-year trends in cholangiocarcinoma incidence in the U.S.: intrahepatic disease on the rise. Oncologist.

[12] Vithayathil M., Khan S.A. (2022). Current epidemiology of cholangiocarcinoma in Western countries. J Hepatol.

[13] Wang Y., Alsaraf Y., Bandaru S.S. (2024). Epidemiology, survival and new treatment modalities for intrahepatic cholangiocarcinoma. J Gastrointest Oncol.

[14] Izquierdo-Sanchez L., Lamarca A., La Casta A. (2022). Cholangiocarcinoma landscape in Europe: diagnostic, prognostic and therapeutic insights from the ENSCCA registry. J Hepatol.

[15] Kelley R.K., Ueno M., Yoo C. (2023). Pembrolizumab in combination with gemcitabine and cisplatin compared with gemcitabine and cisplatin alone for patients with advanced biliary tract cancer (KEYNOTE-966): a randomised, double-blind, placebo-controlled, phase 3 trial. Lancet.

[16] Bowlus C.L., Arrivé L., Bergquist A. (2023). AASLD practice guidance on primary sclerosing cholangitis and cholangiocarcinoma. Hepatology.

[17] Clements O., Eliahoo J., Kim J.U., Taylor-Robinson S.D., Khan S.A. (2020). Risk factors for intrahepatic and extrahepatic cholangiocarcinoma: a systematic review and meta-analysis. J Hepatol.

[18] Tyson G.L., El-Serag H.B. (2011). Risk factors for cholangiocarcinoma. Hepatology.

[19] Petrick J.L., Yang B., Altekruse S.F. (2017). Risk factors for intrahepatic and extrahepatic cholangiocarcinoma in the United States: a population-based study in SEER-medicare. PLoS One.

[20] European Association for the Study of the Liver (2023). Electronic address: easloffice@easloffice.eu, European Association for the Study of the Liver. EASL-ILCA Clinical Practice Guidelines on the management of intrahepatic cholangiocarcinoma. J Hepatol.

[21] Marzioni M., Maroni L., Aabakken L. (2025). EASL Clinical Practice Guidelines on the management of extrahepatic cholangiocarcinoma. J Hepatol.

[22] Osataphan S., Mahankasuwan T., Saengboonmee C. (2021). Obesity and cholangiocarcinoma: a review of epidemiological and molecular associations. J Hepatobiliary Pancreat Sci.

[23] Blechacz B. (2017). Cholangiocarcinoma: current knowledge and new developments. Gut Liver.

[24] Cho M.S., Kim S.H., Park S.W. (2012). Surgical outcomes and predicting factors of curative resection in patients with hilar cholangiocarcinoma: 10-year single-institution experience. J Gastrointest Surg.

[25] Cillo U., Fondevila C., Donadon M. (2019). Surgery for cholangiocarcinoma. Liver Int.

[26] Oh D.Y., Ruth He A., Qin S. (2022). Durvalumab plus gemcitabine and cisplatin in advanced biliary tract cancer. NEJM Evid.

[27] Macias R.I.R., Kanzaki H., Berasain C., Avila M.A., Marin J.J.G., Hoshida Y. (2025). The search for risk, diagnostic and prognostic biomarkers of cholangiocarcinoma and their biological and clinicopathological significance. Am J Pathol.

[28] Micalizzi D.S., Sequist L.V., Haber D.A. (2024). Deploying blood-based cancer screening. Science.

[29] Karlsen T.H., Rutter H., Carrieri P. (2024). The EASL-Lancet commission on liver health in Europe: prevention, case-finding, and early diagnosis to reduce liver-related mortality. Lancet.

[30] Muñoz-Martínez S., Rimola J., Londoño M.C., Cárdenas A., Forner A. (2022). Cholangiocarcinoma: early detection and screening in high-risk population. Hepatoma Res.

[31] National Institutes of Health All of Us Reasearch Program. https://allofus.nih.gov.

[32] Verma A., Damrauer S.M., Naseer N. (2022). The Penn Medicine BioBank: towards a genomics-enabled learning healthcare system to accelerate precision medicine in a diverse population. J Pers Med.

[33] Palchuk M.B., London J.W., Perez-Rey D. (2023). A global federated real-world data and analytics platform for research. JAMIA Open.

[34] UK Biobank https://www.ukbiobank.ac.uk/.

[35] Heidari S., Babor T.F., De Castro P., Tort S., Curno M. (2016). Sex and gender equity in research: rationale for the SAGER guidelines and recommended use. Res Integr Peer Rev.

[36] Wang G., Heij L.R., Liu D. (2022). The role of single-nucleotide polymorphisms in cholangiocarcinoma: a systematic review. Cancers (Basel).

[37] Lampropoulou D.I., Laschos K., Aravantinos G. (2021). Association between homeobox protein transcript antisense intergenic ribonucleic acid genetic polymorphisms and cholangiocarcinoma. World J Clin Cases.

[38] Sjoberg D.D., Whiting K., Curry M., Lavery J.A., Larmarange J. (2021). The R Journal: Reproducible Summary Tables with the gtsummary Package.

[39] Pedregosa F., Varoquaux G., Gramfort A. (2011). Scikit-learn: machine learning in python. J Mach Learn Res.

[40] CatBoost. https://catboost.ai/docs/en/.

[41] Youden W.J. (1950). Index for rating diagnostic tests. Cancer.

[42] scikit-learn fbeta_score. https://scikit-learn.org/stable/modules/generated/sklearn.metrics.fbeta_score.html.

[43] Rodríguez-Pérez R., Bajorath J. (2020). Interpretation of machine learning models using shapley values: application to compound potency and multi-target activity predictions. J Comput Aided Mol Des.

[44] Lundberg S., Lee S.I. (2017). Advances in neural information processing systems 30 (NeurIPS 2017).

[45] Kerr K.F., Brown M.D., Zhu K., Janes H. (2016). Assessing the clinical impact of risk prediction models with decision curves: guidance for correct interpretation and appropriate use. J Clin Oncol.

[46] Baker S.G. (2009). Putting risk prediction in perspective: relative utility curves. J Natl Cancer Inst.

[47] Penn medicine BioBank - Home. https://pmbb.med.upenn.edu/.

[48] Data snapshots. https://www.researchallofus.org/data-tools/data-snapshots/.

[49] Survey explorer. https://www.researchallofus.org/data-tools/survey-explorer/.

[50] Biobank. https://allofus.nih.gov/article/biobank.

[51] Nagai K., Tanaka T., Kodaira N., Kimura S., Takahashi Y., Nakayama T. (2021). Data resource profile: JMDC claims database sourced from health insurance societies. J Gen Fam Med.

[52] 株式会社JMDC (2021). https://www.jmdc.co.jp/en/jmdc-claims-database/.

[53] Banales J.M., Cardinale V., Carpino G. (2016). Expert consensus document: cholangiocarcinoma: current knowledge and future perspectives consensus statement from the European Network for the Study of cholangiocarcinoma (ENS-CCA). Nat Rev Gastroenterol Hepatol.

[54] Collins G.S., Reitsma J.B., Altman D.G., Moons K.G.M. (2015). Transparent reporting of a multivariable prediction model for individual prognosis or diagnosis (TRIPOD): the TRIPOD statement. BMJ.

[55] Shen W., Ning J., Yuan Y. (2015). A direct method to evaluate the time-dependent predictive accuracy for biomarkers: a direct method to evaluate the time-dependent predictive accuracy. Biometrics.

[56] All of Us Research Program protocol. https://allofus.nih.gov/article/all-us-research-program-protocol.

[57] Bycroft C., Freeman C., Petkova D. (2018). The UK Biobank resource with deep phenotyping and genomic data. Nature.

[58] Ferber D., El Nahhas O.S.M., Wölflein G. (2025). Development and validation of an autonomous artificial intelligence agent for clinical decision-making in oncology. Nat Cancer.

[59] Chen H., Ma X., Rives H., Serpedin A., Yao P., Rameau A. (2024). Trust in machine learning driven clinical decision support tools among otolaryngologists. Laryngoscope.

[60] Wray N.R., Ripke S., Mattheisen M. (2018). Genome-wide association analyses identify 44 risk variants and refine the genetic architecture of major depression. Nat Genet.

[61] Cross A.J., Moore S.C., Boca S. (2014). A prospective study of serum metabolites and colorectal cancer risk: metabolites and colorectal cancer. Cancer.

[62] Shu X., Zheng W., Yu D. (2018). Prospective metabolomics study identifies potential novel blood metabolites associated with pancreatic cancer risk: prospective metabolomics study. Int J Cancer.

[63] Whitfield J.B. (2001). Gamma glutamyl transferase. Crit Rev Clin Lab Sci.

[64] Gellert-Kristensen H., Bojesen S.E., Tybjærg Hansen A., Stender S. (2024). Telomere length and risk of cirrhosis, hepatocellular carcinoma, and cholangiocarcinoma in 63,272 individuals from the general population. Hepatology.

[65] Gerussi A., Bernasconi D.P., O’Donnell S.E. (2021). Measurement of gamma glutamyl transferase to determine risk of liver transplantation or death in patients with primary biliary cholangitis. Clin Gastroenterol Hepatol.

[66] Krishnan A., Schneider C.V., Arkenau H.T. (2024). Association between incretin-based drugs and risk of cholangiocarcinoma among patients with type 2 diabetes: a large population-based matched cohort study. J Clin Transl Endocrinol.

[67] Saengboonmee C., Seubwai W., Lert-Itthiporn W., Sanlung T., Wongkham S. (2021). Association of diabetes mellitus and cholangiocarcinoma: update of evidence and the effects of antidiabetic medication. Can J Diabetes.

[68] Qin S.S., Pan G.Q., Meng Q.B., Liu J.B., Tian Z.Y., Luan S.J. (2023). The causal relationship between metabolic factors, drinking, smoking and intrahepatic cholangiocarcinoma: a Mendelian randomization study. Front Oncol.

[69] Huang Y., You L., Xie W., Ning L., Lang J. (2017). Smoking and risk of cholangiocarcinoma: a systematic review and meta-analysis. Oncotarget.

[70] Baidoun F., Sarmini M.T., Merjaneh Z., Moustafa M.A. (2022). Controversial risk factors for cholangiocarcinoma. Eur J Gastroenterol Hepatol.

[71] Park J.H., Hong J.Y., Han K. (2023). Association between smoking cessation and the risk of cholangiocarcinoma and ampulla of vater cancer: a nationwide cohort study. Liver Cancer.

[72] Huber Y., Bierling F., Labenz C. (2018). Validation of insulin-like growth factor-1 as a prognostic parameter in patients with hepatocellular carcinoma in a European cohort. BMC Cancer.

[73] Schneider C.V., Gross S., Balasubramani S. (2025). Insulin-like growth Factor-1 reflects liver disease stage and improves prediction of liver-related mortality. Clin Gastroenterol Hepatol.

[74] Min-Oo H.H., Aung T.M., Wongwattanakul M., Maraming P., Proungvitaya T., Proungvitaya S. (2023). Development of a prognostic score for cholangiocarcinoma patients using a combination of biochemical parameters. In Vivo.

[75] Clusmann J., Koop P.H., Zhang D.Y. (2026). Machine learning predicts hepatocellular carcinoma risk from routine clinical data: a large population-based multicentric study. Cancer Discov.

[76] Kwo P.Y., Cohen S.M., Lim J.K. (2017). ACG clinical guideline: evaluation of abnormal liver chemistries. Am J Gastroenterol.

[77] Newsome P.N., Cramb R., Davison S.M. (2018). Guidelines on the management of abnormal liver blood tests. Gut.

[78] McDermott M.B.A., Zhang H., Hansen L.H., Angelotti G., Gallifant J. (2024). Advances in neural information processing systems 37 (NeurIPS 2024).

[79] Van Calster B., Nieboer D., Vergouwe Y., De Cock B., Pencina M.J., Steyerberg E.W. (2016). A calibration hierarchy for risk models was defined: from utopia to empirical data. J Clin Epidemiol.

