# Supplementary material for: Machine learning for population-level risk prediction of future cholangiocarcinoma
Source: eBioMedicine. 2026 Aug 12;131:106433. doi: 10.1016/j.ebiom.2026.106433 (PMC13487892; doi:10.1016/j.ebiom.2026.106433)
Supplement: Supplementary file 1 [file mmc1.docx]

Supplementary Material to

**Machine learning for population-level risk prediction of future cholangiocarcinoma**

Felix van Haag (1, ‡), Jan Clusmann, MD (1, 2, 3, 4, ‡), Paul-Henry Koop, MD (2, 3, 4), Ryan Goodson (5), Ilya Tryakin (3), Shun-Ichi Wakabayashi, MD, PhD (6), Tobias Seibel (1), Niharika Jakhar (1), Helen Ye Rim Huang (1, 7), David Y. Zhang (8, 9), Inuk Zandvakili, MD, PhD (8, 10), Arunkumar Krishnan, MD, MSc (11), Pedro M. Rodrigues, PhD (12), Jesus M. Banales, PhD (12, 13) , Alessio Gerussi, MD, PhD (14, 15), Anna Saborowski, MD (16), Daniel Rader, MD (7, 8), Jakob N. Kather, MD, MSc (2, 3, 17, 18), Kai Markus Schneider, MD, PhD (1, 2, 3, 19) , Carolin Victoria Schneider, MD (1, 2, 3,*)

1. Department of Internal Medicine III, Gastroenterology, Metabolic Diseases and Intensive Care, University Hospital RWTH Aachen, Aachen, Germany
2. Department of Medicine I, Faculty of Medicine and University Hospital Carl Gustav Carus, TUD Dresden University of Technology, Dresden, Germany
3. Else Kroener Fresenius Centre for Digital Health, Faculty of Medicine and University Hospital Carl Gustav Carus, TUD Dresden University of Technology, Dresden, Germany
4. German Cancer Consortium (DKTK) partner site Dresden and German Cancer Research Centre (DKFZ), Heidelberg, Germany
5. University of Cincinnati, College of Medicine, Cincinnati OH, 45267 USA
6. Division of Gastroenterology, Department of Medicine, Shinshu University School of Medicine, Nagano, Japan
7. The Perelman School of Medicine, The Institute for Translational Medicine and Therapeutics, University of Pennsylvania, Philadelphia, Pennsylvania, USA
8. Department of Medicine, University of Pennsylvania Perelman School of Medicine, Philadelphia, PA, 19104, USA
9. Department of Genetics, University of Pennsylvania Perelman School of Medicine, Philadelphia, PA, 19104, USA
10. Division of Gastroenterology and Hepatology, Department of Internal Medicine, College of Medicine, University of Cincinnati, Cincinnati, Ohio; Division of Gastroenterology and Hepatology, Department of Medicine, University of Pennsylvania, Philadelphia, Pennsylvania
11. Department of Supportive Oncology, Atrium Health Levine Cancer, Charlotte, NC 28204, USA
12. Department of Liver and Gastrointestinal Diseases, Biogipuzkoa Health Research Institute, Donostia University Hospital, University of the Basque Country (UPV/EHU), Ikerbasque, CIBERehd, San Sebastian, Spain
13. Department of Biochemistry and Genetics, School of Sciences, University of Navarra, Pamplona, Spain.
14. Department of Medicine and Surgery, University of Milano-Bicocca, Monza, Italy;
15. Centre for Autoimmune Liver Diseases & Division of Gastroenterology, Fondazione IRCCS San Gerardo dei Tintori, ERN RARE LIVER, Monza, Italy
16. Department of Gastroenterology, Hepatology, Infectious Diseases and Endocrinology. Hannover Medical School, Hannover, Germany
17. Medical Oncology, National Centre for Tumor Diseases (NCT), University Hospital Heidelberg, Heidelberg, Germany
18. Pathology & Data Analytics, Leeds Institute of Medical Research at St James's, University of Leeds, Leeds, United Kingdom
19. Centre for Regenerative Therapies Dresden (CRTD), Technische Universität (TU), Dresden, Germany

‡ Contributed equally

*** Corresponding author:** cschneider@ukaachen.de

Carolin Victoria Schneider, MD, Professor of Prevention and Genetics of metabolic diseases of the liver

Department of Medicine III

University Hospital RWTH Aachen

DE– 52074 Aachen

Phone: +49 241 80 866

Mail: cschneider@ukaachen.de

ORCID ID: 0000-0002-6728-9246

# **Supplementary Figures**

### Supplementary Figure 1 - Data flowchart

Flowchart of data included in our study, with N = number of all participants included and n = participants with a diagnosis of cholangiocarcinoma; the Train/Test and Validation split (80%/20%) was performed on all CCA cohorts.

### Supplementary Figure 2 - Model Performance for intrahepatic vs extrahepatic CCA

Curves and plots shown for incremental Models A-E for the Random Forest Classifier, respectively. (A) / (B) Violin plots for prediction values of cases and controls for iCCA-All (A) and eCCA-All (B) for the RFC Model; Dashed and dotted lines represent 50^th^, as well as 25^th^ and 75^th^ percentiles, respectively. (C) / (D) Receiver operating characteristics curves for iCCA-All (C) and eCCA-All (D) for Random Forest Classifier (RFC); Area under the curve values depicted in the box for each curve; dashed line represents AUC of 0.5 (E) / (F) Precision Recall Curves for iCCA-All (E) and eCCA-All (F) for RFC; Zoomed in view in the middle; Area under the curve values depicted in the box for each curve.

### Supplementary Figure 3 - Feature importance and SHAP analysis for CCA-Unscreened and CCA-PAR

(A)/(B) Random Forest feature importance bar plots for CCA-Unscreened Model C (A) and Model E (B); top 20 features ordered by importance for model prediction, bar colours depict group association with mean feature importance by group shown in lower-right box with 95% CI (grey bars). (C)/(D) SHapley Additive ePlanation (SHAP) beeswarm plot with top 10 features for RFC CCA-Unscreened Model C (C) and Model E (D); Colours indicate direction of feature value (blue = low, red = high). (E)/(F) as in A/B, but for CCA-PAR Model C (E) and Model E (F) (G)/(H) as in C/D, but for RFC CCA-Unscreened Model C (G) and Model E (H). Gamma GT: Gamma Glutamyltransferase; BMI: Body Mass Index; IGF-1: Insulin-like Growth Factor 1; MCV: Mean Corpuscular Volume; DM: Diabetes mellitus; TFA: Total Fatty Acids; MUFA: Mono Unsaturated Fatty Acids; PUFA: Poly Unsaturated Fatty Acids; CE: Cholesteryl Esters; HDL: High Density Lipoproteins; LDL: Low Density Lipoproteins; SHBG: Sex hormone binding globulin

### Supplementary Figure 4 - Model ablation performance

(A) Receiver operation characteristics curves (AUROC) for Models C, TOP75, TOP30, TOP20, TOP10, TOP5 and TOP4 for RFC (dotted) in UKB, with area under the curve values in the bottom right corner. Dashed grey line depicts AUC of 0.5. (B) Area under Precision Recall curves (AUPRC) for Model C, TOP75, TOP30, TOP20, TOP10, TOP5 and TOP4 for RFC (dotted) in UKB with area under the curve values in the top right corner.

### Supplementary Figure 5 - External validation of Model TOP4 in UKB test set, PMBB, AOU and JMDC

(A) Receiver operation characteristics curves (AUROC) for Model TOP4 for RFC (dotted) and CatBoost (line) in UKB (red), Penn Medicine BioBank (PMBB) (blue) and All of Us (AOU) (green) cohorts, with area under the curve values in the bottom right corner. Dashed grey line depicts AUC of 0.5 (B) (B) Precision recall curves (PRC) for Model TOP4 for RFC (dotted) and CatBoost (line) in UKB (red), PMBB (blue) and AOU (green) cohorts; Area under the curves values in the upper right corner. (C) Confusion matrices for CatBoost UKB, PMBB, AOU and JMDC cohorts from left to right for different thresholds depicted, PPV = Positive predictive value. NPV = Negative predictive value. Colouring indicates relative percentage per class.PMBB cohort for the validation of the TOP4 is different to validation of the TOP5 Model, interpret and compare with caution.

### Supplementary Figure 6 - Odds ratios for Group comparison in the TriNetX cohort

On the left, control groups (rectangles) and experimental groups (triangles) are defined by inclusion criteria based on the TOP5 model (see External Validation/TriNetX). The forest plot on the right displays odds ratios (ORs) for each comparison with 95% confidence intervals at each side; Sex is indicated by colour (male: turquoise; female: orange), and the size of each point reflects the number of CCA cases in the experimental group. In the comparison between the healthy group and the “5 of 5” group, no matched cases remained in the control group, rendering OR calculation infeasible. GGT: Gamma glutamyltransferase; Feat.: Feature

**Supplementary Figure 7 - Numbers needed to screen per threshold and recall in UKB, AOU and JMDC**

(A) Recall (sensitivity) and Number needed to screen (NNS) for CatBoost Models TOP5 at various thresholds in the UKB independent test set (upper graph), the AOU external validation (middle graph) and JMDC external validation (lower graph). (B) Corresponding Recall/NNS relations for CatBoost TOP4 Model in the UKB, AOU and JMDC cohorts; Colours indicating lower/higher threshold values.

**Supplementary Figure 8 - Model TOP5 Calibration evaluation on the UKB cohort**

Results shown in this figure are displayed for CatBoost Model TOP5 (a) Predicted risk distribution before calibration in the UKB cohort (b) Predicted risk distribution after calibration in the UKB cohort (c) Calibration curve before calibration (d) Calibration curve after calibration (e) Threshold dependent positive predictive value distribution before and after calibration in the UKB cohort

**Supplementary Figure 9 - Model TOP5 Calibration evaluation on the AOU cohort**

Results shown in this figure are displayed for CatBoost Model TOP5 (a) Predicted risk distribution before calibration in the AOU cohort (b) Predicted risk distribution after calibration in the AOU cohort (c) Calibration curve before calibration (d) Calibration curve after calibration (e) Threshold dependent positive predictive value distribution before and after calibration in the AOU cohort

### Supplementary Figure 10: Decision curve analysis for the TOP5-CatBoost model in the UKB cohort

Clinical net benefit (left y-axis) and standardised net benefit (right y-axis) are shown before and after model calibration, compared against the default "Treat All" and "Treat None" strategies. Evaluation was performed on the UKB holdout cohort (n = 91,056, CCA = 82). The labeled point highlights the recommended clinical cutoff at a 0.6% probability threshold for maximum net benefit.

# **Table of content for supplementary tables (*See Supplementary_Material.xlsx*)**

| **Supplementary Table Number** | **Legend** |
| --- | --- |
| **Supplementary Table 1** | **Covariate Features taken at assessment** |
| **Supplementary Table 2** | **ICD10 codes used as single-entity diagnosis features** |
| **Supplementary Table 3** | **ICD10 codes used as grouped diagnosis features** |
| **Supplementary Table 4** | **Diagnoses comparing CCA to non-CCA group, split by sex** |
| **Supplementary Table 5** | **Blood count features from UKB included in training** |
| **Supplementary Table 6** | **Blood features comparing CCA to non-CCA, split by sex** |
| **Supplementary Table 7** | **single nucleotide polymorphisms (SNPs) included as features in training, with reference attached** |
| **Supplementary Table 8** | **Metabolomic features from UKB included in training** |
| **Supplementary Table 9** | **Metabolomics features comparing CCA to non-CCA group, split by sex** |
| **Supplementary Table 10** | **Centre splits** |
| **Supplementary Table 11** | **CCA cases per centre** |
| **Supplementary Table 12** | **Missingness rates for the UKB features** |
| **Supplementary Table 13** | **Grid search options** |
| **Supplementary Table 14** | **Final Hyperparameter settings for all models** |
| **Supplementary Table 15** | **Diagnoses included in Patients at risk (PAR) cohort** |
| **Supplementary Table 16** | **Diagnoses excluded from Unscreened cohort** |
| **Supplementary Table 17** | **DeLong tests for CCA-ALL cohort** |
| **Supplementary Table 18** | **DeLong tests for CCA-PAR cohort** |
| **Supplementary Table 19** | **DeLong tests for CCA-Unscreened cohort** |
| **Supplementary Table 20** | **DeLong tests for estimator comparison** |
| **Supplementary Table 21** | **DeLong tests for feature ablation models** |
| **Supplementary Table 22** | **Evaluation metrics for model performance threshold independent for RFC** |
| **Supplementary Table 23** | **Evaluation metrics for model performance threshold dependent for RFC** |
| **Supplementary Table 24** | **Evaluation metrics for model performance threshold dependent for CatBoost** |
| **Supplementary Table 25** | **Evaluation metrics for model performance threshold independent for CatBoost** |
| **Supplementary Table 26** | **Feature reduction for ablation study** |
| **Supplementary Table 27** | **Time dependent performance evaluation** |
| **Supplementary Table 28** | **UKB-PMBB data mappings** |
| **Supplementary Table 29** | **PMBB cohort demographic data comparing CCA to non-CCA, split by sex** |
| **Supplementary Table 30** | **UKB - AOU name mappings** |
| **Supplementary Table 31** | **UKB - AOU data mappings** |
| **Supplementary Table 32** | **AOU cohort diagnoses data comparing CCA to non-CCA, split by sex** |
| **Supplementary Table 33** | **AOU cohort blood data comparing CCA to non-CCA, split by sex** |
| **Supplementary Table 34** | **TriNetX group comparison** |
| **Supplementary Table 35** | **TriNetX Demographic data** |
| **Supplementary Table 36** | **JMDC Demographic data** |
| **Supplementary Table 37** | **Calibration Metrics AOU cohort** |
